# The flowering transition pathways converge into a complex gene regulatory network that underlies the phase changes of the shoot apical meristem in *Arabidopsis thaliana*

**DOI:** 10.3389/fpls.2022.852047

**Published:** 2022-08-09

**Authors:** Elva C. Chávez-Hernández, Stella Quiroz, Berenice García-Ponce, Elena R. Álvarez-Buylla

**Affiliations:** ^1^Laboratorio de Genética Molecular, Desarrollo y Evolución de Plantas, Departamento de Ecología Funcional, Instituto de Ecología, Universidad Nacional Autónoma de México, Mexico City, Mexico; ^2^Centro de Ciencias de la Complejidad, Universidad Nacional Autónoma de México, Mexico City, Mexico

**Keywords:** gene regulatory network, flowering, shoot apical meristem, XAANTAL2, Boolean model, phase transitions

## Abstract

Post-embryonic plant development is characterized by a period of vegetative growth during which a combination of intrinsic and extrinsic signals triggers the transition to the reproductive phase. To understand how different flowering inducing and repressing signals are associated with phase transitions of the Shoot Apical Meristem (SAM), we incorporated available data into a *dynamic* gene regulatory network model for *Arabidopsis thaliana*. This Flowering Transition Gene Regulatory Network (FT-GRN) formally constitutes a *dynamic* system-level mechanism based on more than three decades of experimental data on flowering. We provide novel experimental data on the regulatory interactions of one of its twenty-three components: a MADS-box transcription factor XAANTAL2 (XAL2). These data complement the information regarding flowering transition under short days and provides an example of the type of questions that can be addressed by the FT-GRN. The resulting FT-GRN is highly connected and integrates developmental, hormonal, and environmental signals that affect developmental transitions at the SAM. The FT-GRN is a *dynamic* multi-stable Boolean system, with 2^23^ possible initial states, yet it converges into only 32 attractors. The latter are coherent with the expression profiles of the FT-GRN components that have been experimentally described for the developmental stages of the SAM. Furthermore, the attractors are also highly robust to initial states and to simulated perturbations of the interaction functions. The model recovered the meristem phenotypes of previously described single mutants. We also analyzed the attractors landscape that emerges from the postulated FT-GRN, uncovering which set of signals or components are critical for reproductive competence and the time-order transitions observed in the SAM. Finally, in the context of such GRN, the role of XAL2 under short-day conditions could be understood. Therefore, this model constitutes a robust biological module and the first multi-stable, *dynamical* systems biology mechanism that integrates the genetic flowering pathways to explain SAM phase transitions.

## Introduction

In plants, new organs develop post-embryonically from the apical meristems, where the progeny of a relatively small group of pluripotent stem cells proliferate at the shoot apex and at the root tips (Kaufmann et al., 2010a). After a period of vegetative growth, plants respond to a combination of intrinsic signals such as aging, hormones, and carbohydrates, as well as environmental cues like photoperiod and temperature, to trigger the transition to the reproductive phase (Quiroz et al., 2021). Both types of signals are perceived in the leaves and at the shoot apical meristem (SAM), although phase transitions occur at the latter. In *Arabidopsis thaliana*, the SAM undergoes two main phase changes, one within the vegetative state and another to the reproductive state. In the first one, the juvenile vegetative meristem (JVM) changes to the mature vegetative stage (Huijser and Schmid, 2011). Afterward, the adult vegetative meristem (AVM) becomes the inflorescence meristem (IM) that can form floral meristems (FM) at its flanks (Kaufmann et al., 2010a). The FM later on sub-differentiates into sepal, petal, stamen, and carpel primordia, while the IM remains undifferentiated during the rest of the plant’s life (Alvarez-Buylla et al., 2010; Posé et al., 2012).

More than three decades of research have uncovered many molecular components involved in the perception of signals that trigger flowering in *A. thaliana*. Epistasis assays and molecular analyses had been used to classify the genetic components into four genetic “*flowering pathways*”: the long-day (LD) photoperiod pathway that relies on CONSTANS (CO) and FLOWERING LOCUS T (FT) proteins; the gibberellin (GA) signaling pathway which generally ubiquitinates DELLA proteins to liberate GA responsive transcription factors (TFs); the vernalization pathway that includes the epigenetic silencing of the flowering repressor *FLOWERING LOCUS C (FLC)* after a long period of cold; and the photoperiod-autonomous pathway where several proteins like FLOWERING CONTROL LOCUS A (FCA), FLOWERING LOCUS D (FLD), FLOWERING LOCUS KH DOMAIN (FLK), FPA, FVE, FY and LUMINIDEPENDENS (LD), among others, repress *FLC* expression under optimal temperature conditions by epigenetic and RNA-processing mechanisms (Srikanth and Schmid, 2011).

Other flowering pathways have emerged describing components responsive to aging, carbohydrate status, and suboptimal temperatures (Blázquez et al., 2003; Wang et al., 2009; Kumar et al., 2012; Wahl et al., 2013). In the aging pathway, the microRNA156 (miR156) represses members of the *SQUAMOSA PROMOTER BINDING PROTEIN LIKE* (*SPL*) family, as well as the miR172 that targets, *APETALA2* (*AP2*) and *AP2*-like genes including, *TARGET OF EAT* (*TOE*)*1-3, SCHLAFMÜTZE* (*SMZ*), and *SCHNARCHZAPFEN* (*SNZ*) involved in developmental phase decisions (Wang et al., 2009; Wu et al., 2009). The production of trehalose-6-phosphate (T6P) by the T6P SYNTHASE 1 (TPS1) serves to detect the carbohydrate status to induce flowering transition (Van Dijken et al., 2004; Wahl et al., 2013). In contrast, genes such as *SHORT VEGETATIVE PHASE* (*SVP*) and the *MADS AFFECTING FLOWERING 1-4 (MAF1-4)* are part of the thermo-sensory pathway (Lee et al., 2013; Posé et al., 2013). Additionally, it has been found that *PHYTOCHROME INTERACTING FACTORS* (*PIF*s) participate in high temperature flowering regulation (Kumar et al., 2012).

Genetic analyses have also led to the discovery of genes that respond to multiple signals. For example *SUPPRESSOR OF OVEREXPRESSION OF CONSTANS (SOC1)* expression is regulated by vernalization, aging, gibberellins and a long-day photoperiod (Lee and Lee, 2010). Therefore, *SOC1, FT*, and *LEAFY (LFY)* were named floral pathway integrators, considering their capacity to respond to different cues and transduce those signals to the floral meristem identity genes: *LFY*, *APETALA1* (*AP1*), and *CAULIFLOWER* (*CAL*; Posé et al., 2012). The advancement of genome-wide analysis by high-throughput technologies resulted in a conceptual change from the view of linear and parallel pathways of flowering transition into an integrated genetic network perspective similar to that used to understand other developmental processes (Pajoro et al., 2014a; Álvarez-Buylla et al., 2016). However, a formal system-level view that integrates all the pathways discovered so far, that regulate flowering and SAM phase transitions in response to multiple stimuli is still lacking.

Experimentally, it is difficult to assess the integration of different signals perceived by plants as they transit into the reproductive phase. GRN *dynamic* modeling using systems biology approaches and computational tools can help us understand, integrate, describe and predict complex biological processes with formal and system-level mechanistic approaches (Azpeitia et al., 2014). Also, they help us to postulate novel hypotheses or predictions not yet experimentally proven. Furthermore, mathematical modeling is instrumental for understanding how the GRN architecture and *dynamics* restrain the evolution of developmental and morphological patterns (Périlleux et al., 2014; Azpeitia et al., 2021). In this sense, ordinary differential equations have been used to elaborate continuous models that predict flowering time (Jaeger et al., 2013; Wang et al., 2014; Leal Valentim et al., 2015; van Dijk and Molenaar, 2017; Haspolat et al., 2019). Although, on those models, flowering regulation is restricted to long-day photoperiod signals and they rely mainly on the integrator genes. Furthermore, GRN Boolean or discrete models have been useful for understanding early floral organ specification (Espinosa-Soto et al., 2004). On the other hand, a Boolean model combined with genetic programming algorithms, predicted alternative regulatory interactions of six genes involved in the flowering transition in response to a long-day photoperiod (Dinh et al., 2017).

More recently, a discrete multivalued model explained the role of *XAANTAL2 (XAL2)* in flowering transition. Furthermore, it explained the paradoxical phenotype of the overexpression line which develops flowers with vegetative traits, while it shows an early flowering phenotype. XAL2 is one of the few known transcriptional factors that positively regulate *TERMINAL FLOWER 1* (*TFL1*; Pérez-Ruiz et al., 2015; Azpeitia et al., 2021), which is essential in IM identity, but it also delays flowering (Shannon and Meeks-Wagner, 1991; Ratcliffe et al., 1998). The model provided a mechanistic explanation that solves this paradox and recovered the VM, the IM and the FM attractors in response to LD photoperiod signaling (Pérez-Ruiz et al., 2015). However, when that model was proposed, information regarding XAL2’s function under non-inductive flowering conditions was lacking even though the strongest flowering phenotype of the *xal2* mutant is observed when plants are grown under a short-day (SD) photoperiod.

We postulate that a larger GRN regulatory module should incorporate the necessary and sufficient components and interactions, so that endogenous and environmental signals involved could be modeled simultaneously. Hence, observed phenotypes of *A. thaliana* wild-type and mutant lines under both photoperiods could be explained, including *XAL2* loss- and gain of function mutants. To approach this hypothesis, we integrated most of the current knowledge on flowering transition into a formal multi-stable *dynamic* Boolean network model. Thus, we gathered and integrated data from over three decades of research to postulate a single multi-stable GRN in which the *“flowering pathways”* unite. We decided to use a Boolean approach to study the *dynamics* of the complex interactions among the diverse components of the system (genes, RNAs, proteins, and hormones), given the complexity of the GRN involved in reproductive phase transition which involves over 300 genes (Bouche et al., 2015), and acknowledge the success and usefulness of such approach in previous similar studies (Ortiz-Gutiérrez et al., 2015; García-Gómez et al., 2017, 2020).

In a Boolean model, the network nodes, which represent the components of the system, are binary variables that evolve over discrete time steps, meaning that the components can be either absent or inactive (0), or active (1) depending on the logic functions that describe the concerted action of its regulators at a previous time step (Kauffman, 1969). Therefore, GRN Boolean models are systemic (relating to a system composed of the nodes and their interactions), *dynamic* (because they evolve in time), and formal (they use logic-based mathematical statements for the nodes interactions) descriptions of a GRN. This type of model captures the logic behind non-linear regulatory relationships without kinetic parameters, which are difficult to obtain experimentally. Previous studies have shown that it is the qualitative nature of the logical functions, rather than the kinetic details, which determine the overall *dynamics* of the network (Sánchez-Corrales et al., 2010). Boolean-GRN systems attain self-sustained steady states, known as attractors, which can be interpreted as the gene expression configurations or system states correlated to cellular types (Kauffman, 1992). Thus, Boolean GRN models are useful to study cell-type differentiation in both plant and animal systems (Huang et al., 2009; Azpeitia et al., 2014; Abou-Jaoudé et al., 2016).

In this work, we present a Flowering Transition GRN (FT-GRN) Boolean model that includes, for the first time, many of the factors known to be essential in developmental, and environmental signaling during floral transition. Our model constitutes a multi-stable system because it recovers more than one steady states with the gene expression patterns found at the SAM during juvenile and adult vegetative phases, and the transition to the reproductive state. The FT-GRN incorporates previous and novel experimental data on the developmental role of *XAL2* during flowering transition in response to age, GA signaling, and a LD photoperiod. The *dynamic* model was validated with *in silico* analyses of single mutants and random perturbations. We also explored the attractors landscape that emerges from the FT-GRN to get insights into the mechanisms guiding the developmental trajectories during the vegetative and reproductive transitions. The FT-GRN constitutes a multi-stable, *dynamical* systems biology mechanism that underlies SAM phase transitions essential to understand the emergent properties of this complex developmental process.

## Materials and methods

### Experimental procedures

*Arabidopsis thaliana* wild-type and mutant plants used in this study were in the Col-0 background, with the exception of the *gai-1* mutant (NW63; Koornneef et al., 1985) that was in L*er* background. The mutants *sly1-11* (N868440), *spl3-1* (FLAG_173C12; Wu and Poethig, 2006), *spl9-4* (SAIL_150_B05; Wu et al., 2009) and *spl15-1* (SALK_074426; Wu et al., 2009), correspond to T-DNA insertion lines while the *xal2-2* mutant has a *En1* transposon insertion (Garay-Arroyo et al., 2013). *gai-1* and *sly1-11* were provided from the Nottingham Arabidopsis Stock Centre.

Plants were grown on soil (Sunshine Mix 3 Sungro) under SD photoperiod (9 h light/15 h dark) at 22°C, and GA_3_ treatment was performed as described in Pérez-Ruiz et al. (2015). The entire aerial tissue was collected for semi-quantitative RT-PCRs, while meristem enriched shoot apices were used for RT-qPCRs. All RT-qPCR assays were performed with 3–4 biological replicates (8 plants each) of 26, 36, or 45 days after sowing (das) plants, depending on the experiment.

RNA was extracted with the Direct-zol RNA MiniPrep kit (Zymo Research, United States) and cDNA was obtained with the SuperScript III RT (Invitrogen, Germany). qPCRs were performed in a StepOnePlus thermocycler (Applied Biosystems, United Kingdom) with primers enlisted in Supplementary Table 1. Relative accumulation was obtained as in Pérez-Ruiz et al. (2015) using *PDF2*, *RNAH*, and *UPL7* as internal controls.

### Flowering transition gene regulatory network

The flowering transition gene regulatory network (FT-GRN) was built based on an exhaustive search in the scientific literature. The genes were selected based on the most comprehensive reviews at the time (Srikanth and Schmid, 2011; Andrés and Coupland, 2012), and on the published *dynamic* models of flowering time, SAM transitions and flower development (Espinosa-Soto et al., 2004; Jaeger et al., 2013; Wang et al., 2014; Leal Valentim et al., 2015; Pérez-Ruiz et al., 2015; Dinh et al., 2017). Data supporting the FT-GRN is available in Supplementary Tables 2, 3.

Network topological properties were calculated with the igraph R package. For visualization yEd graph editor was used.

### Boolean model

A Boolean model was used to formalize the FT-GRN interactions and study its *dynamics*. In short, the state of a gene *i* (*x*_*i*_) is equal to 1 if the gene is expressed and its product is active; and *x*_*i*_ = 0 if it is not expressed or is inactive. The system is solved in discrete time steps. The temporal evolution of the state of gene *i* can be represented by:


$$x_{i}\,\left(t+1\right)=F_{i}\,\left(x_{1}\,\left(t\right),x_{2}\,\left(t\right),\ldots ,x_{k}\,\left(t\right)\right)$$

Where the state of gene *i* at time *t+1*, is *x_*i*_(t+1)*, and it is described by a function *F*_*i*_ that depends on the state of its *k* regulators on a previous time step (*t*). Logic functions (Supplementary Table 4) were obtained from reported functional experimental data and also top-down experiments that include expression of reporter lines and RT-PCR in different mutant backgrounds, RNA-seq experiments, chromatin immunoprecipitation (ChIP)-PCR, ChIP-seq and ChIP-ChIP experiments documented in Supplementary Table 3.

For a Boolean system with *n* number of genes, the number of possible states is finite (Ω = 2*^n^*). The *dynamics* of a Boolean network can be well-described by a state transition table. In this table each row represents a state of the Boolean network *a* ∈ {0,1}^*n*^ at time *t* and the corresponding state of the Boolean network *f*(*a*) at time *t* + 1. Therefore, the system will return to a previous visited state and then stay in a cycle of states. This cycle of the states is called an attractor. An attractor is represented by a set of states {*a*_0_,*a*_1_,…,*a*_*p*−1_} where *a*_*i* + 1_ = *f*(*a*_*i*_)(*i*0,…,*p*−2) and *a*_0_ = *f*(*a*_*p*−1_) hold. If *p* = 1, an attractor is called a fixed-point attractor. If *p* > 1, an attractor is called a cyclic attractor. In both cases, *p* is called the period of an attractor. The set of states that will evolve to the same attractor is called the basin of attraction (Akutsu et al., 2008).

The *dynamic* model was solved with both, a synchronous updating scheme, where all nodes are updated at each time step or with a random asynchronous updating scheme, where at each time step a single node is randomly selected to be updated. Simulations with both types of updating schemes were run exhaustively, meaning that all the possible initial states (2^23^) were considered for the simulation. Input nodes were considered as constant. Both schemes gave the same 32 attractors in the FT-GRN model. The *dynamic* analysis of the model was performed with R (R Core Team, 2016) packages BoolNet (Mussel et al., 2010) and BoolNetPerturb (Martínez-Sánchez, 2020). Code for all simulations in this work is available at the repository https://github.com/CaroChavez/FT-GRN (Chavez, 2022).

#### *In silico* attractors and validation *in planta*, with functional and single-cell expression patterns

Since we can understand attractors as the gene expression patterns of a cell type (phenotypes) (Kauffman, 1992), the spatio-temporal expression data (Supplementary Table 5) was compared to the attractors. The genes expression of the network using RNA-seq single-cell data published by Zhang et al. (2021) was downloaded using the http://wanglab.sippe.ac.cn/shootatlas and http://wanglab.sippe.ac.cn/rootatlas/ (Zhang et al., 2019) platforms.

#### *In silico* mutant analysis

For loss-of-function mutants and constitutive expression simulations, the node state was fixed to 0 or 1, respectively. A synchronous updating scheme was used. The relative basin size of each phenotype was calculated as a percentage of the sum of the basin size of the attractors with a specific phenotype with respect to the Ω = 2*^n^* state space, where *n* is the number of nodes.

#### Attractors and state graph robustness to random perturbations

Perturbed networks were constructed by flipping the output value of one row in the truth tables of the model (one bit-flip). Each position of all truth tables (Boolean functions) of the FT-GRN model were consistently altered. Each perturbed network was solved for all possible initial conditions with a synchronous scheme. The attractors and their basin sizes were compared to those of the unperturbed model. For each of the 2*^k^* one bit-flip perturbed models, we classified them as: “identical” if they recovered the same 32 attractors than the unperturbed original model; “subset” if they recovered some of the original attractors but no new ones; or “new” if they recovered new attractors in addition to the original attractors. Then we quantified for each node, the percentage of the 2*^k^* perturbed models that were in these three categories.

Additionally, for each perturbed model we calculated their attractors relative basin sizes as a percentage of all state space. Then, for each node we calculated the mean of new attractor’s relative basin size (NABS) in their perturbed models.

The attractors robustness to random perturbations of the state transition graph was tested in comparison to 1000 alternative random models with the same topological properties. The normalized Hamming distance was used as a measure of dissimilarity between successor states of randomly generated initial states and perturbed copies of these states.

To test which nodes could induce differentiation from an attractor with a given phenotype to another with a different phenotype in response to a transient perturbation, the 32 attractors (Boolean vectors) were taken as initial conditions and then the state of each position in the vector was perturbed by one time step, then we obtained the final steady state and its phenotype.

### Flowering transition gene regulatory network continuous model

The Boolean model was converted to a continuous system using ordinary differential equations (ODE) following the method developed by Mendoza and Xenarios (2006) and Sánchez-Corrales et al. (2010) to automatically translate the topology of a discrete regulatory network into a continuous standardized qualitative *dynamical* system and study its behavior. The Boolean functions were approximated to continuous differentiable sigmoidal functions that are bound between 0 and 1 (Sánchez-Corrales et al., 2010). For each node of the FT-GRN, we had a function *w*_*i*_ that is the continuous form of the truth table describing the state of the node *x*_*i*_. Thus, the logical functions were replaced with a set of continuous functions that satisfy Zadeh’s rules of fuzzy propositional calculus (Zadeh, 1965). The continuous system was modeled as a series of coupled ODE, describing the activation of each node with an equation of the form:


$$\frac{d\,\left(x_{i}\right)}{d\,t}=\frac{-e^{0.5\,h}+e^{-h\,\left(w_{i}-0.5\right)}}{\left(1-e^{0.5\,h}\right)\,\left(1+e^{-h\,\left(w_{i}-0.5\right)}\right)}-\gamma_{i}\,x_{i}$$

The right-hand side of the equation has two parts: a sigmoid function to account for the rate of production and a linear term to account for the decay of *x*_*i*_. The parameter *h* determines the strength of the interactions and controls the curve form: a step function, a logistic function, or a straight line. This sigmoid function was constructed to pass through the points (0, 0), (0.5, 0.5), and (1, 1) for any positive value of *h* (Sánchez-Corrales et al., 2010). The parameter *γ_*i*_* is the decay rate of node *x*_*i*_, and *w*_*i*_ is the continuous form of the Boolean function for node *x_*i*_*, transformed using fuzzy logic following the rules:


q⁢∧⁢p→m⁢i⁢n⁢[q,p],

q∨p→m⁢a⁢x⁢[q,p],

¬⁢p→1-p.

The explicit form of the FT-GRN ODE system including *w*_*i*_ can be found in Chavez (2022). The parameters analysis of the FT-GRN continuous model showed that the model gives similar attractors to the Boolean version for ranges of 10–50 and 0.5–1 for *h* and γ, respectively. Parameters values selected for the simulation were: *h* = 20, *γ_*i*_* = 1. We set the parameter *γ_*i*_* for all nodes equal to 1, so that the expression level of the node at its stationary state is merely determined by the degree of truth of the fuzzy proposition *w*_*i*_. By giving *h* > 0, and *γ_*i*_* ≥ 1, the equations keep the variables *x*_*i*_ in the closed interval [0, 1]. This means that the methodology is designed to describe normalized values of activation, not absolute values of concentration (Mendoza and Xenarios, 2006; Sánchez-Corrales et al., 2010). The FT-GRN continuous model gives gene steady states close to either 0 or 1; the steady states of the system are then comparable to the FT-GRN Boolean model phenotypes. The numerical analysis of the ODE system was conducted using R with packages deSolve (Soetaert and Petzoldr, 2010) and BoolNetPerturb (Martínez-Sánchez, 2020).

#### Attractors landscape exploration

For the attractors landscape analysis we followed Davila-Velderrain et al. (2015b) methodology. The propensity of individual nodes to produce phenotype changes was tested by increasing the decay rate for each individual perturbed node in the continuous FT-GRN model.

For each attractor, and for each active node (*x*_*i*_) in the attractor state: the selected attractor was taken as an initial condition in an ODE system’s initial-value problem; only the decay rate of the selected active node *x*_*i*_ was increased (*γ_*i*_* = 5) while the rest of the parameters of the system were kept constant; then the ODE system was solved numerically until reaching a new steady state. Because the states of the nodes were close to 0 or 1; the steady states of the system were then comparable to the FT-GRN Boolean model phenotypes. The FT-GRN node’s perturbation that changed the phenotype of the initial system state to another phenotype in the final system steady state was recorded.

#### Differentiation in the continuous flowering transition gene regulatory network model

The FT-GRN continuous model was modified by connecting a control node (*u*_*i*_), that targeted one of the FT-GRN inputs as a negative regulator. An initial attractor was taken as the initial condition for the ODE system, and the decay rate of the control element (*u_*i*_)* was increased, which results in an increase of its target. The ODE system was solved numerically until reaching a new steady state.

## Results

### XAL2 is positively regulated by SPL9/15 and mediates gibberellin response in short-day conditions

**FIGURE 1:**
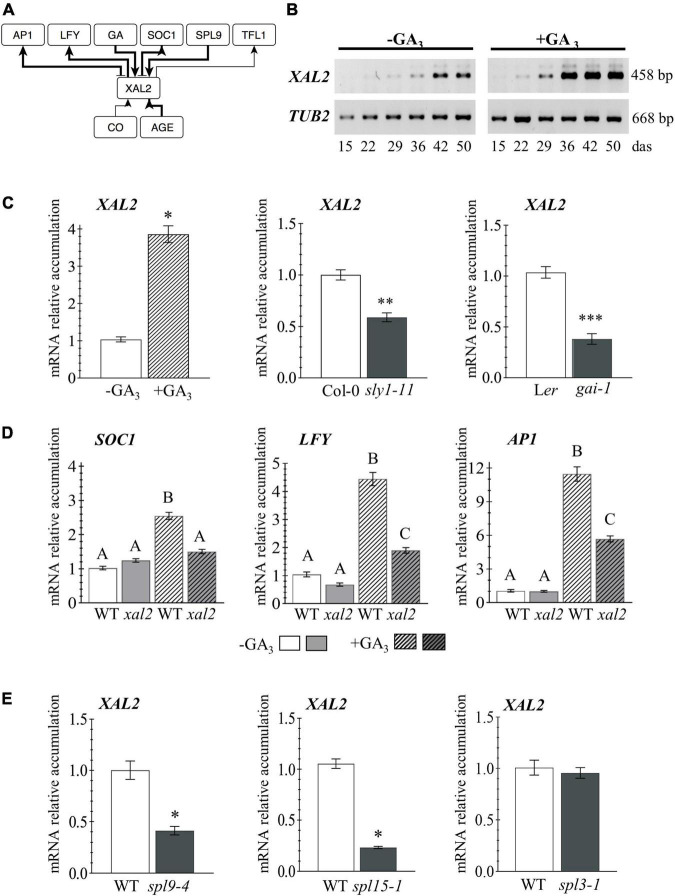
*XAL2* regulation. **(A)** Scheme of *XAL2* regulatory interactions with components of the floral transition GRN under LD and SD conditions. **(B)** *XAL2* mRNA accumulation kinetics in aerial tissues during development and in response to GA_3_ addition. **(C)** *XAL2* is induced by GA_3_ in plants of 26 days after sowing (das) and it is downregulated in two GA signaling mutants at 45 das when endogenous GA levels increase (Eriksson et al., 2006). **(D)** Accumulation of *SOC1* (26 das plants), *LFY*, and *AP1* (36 das plants) mRNA in response to GA_3_ is partially compromised in the *xal2* mutant. **(E)** *XAL2* is positively regulated by SPL9 and SPL15, but not by SPL3 under the same SD conditions (36 das plants). **(B–E)** Plants were grown in SD. For **(C–E)**, RNA was extracted from shoot apices. Data represent the mean value ± standard error. Statistically significant differences in **(C,E)** were confirmed with Student’s *T*-Test (*P < 0.05; ^**^P < 0.01; ^***^P < 0.001), and by One-Way ANOVA (*p* < 0.01), followed by a Holm-Sidak’s Multiple Comparison Test denoted by capital letters in **(D)** (3–4 biological replicates with eight plants each).

Previous work showed that *XAL2* participates in floral transition, specifically, it was established that CO positively regulates *XAL2* while SOC1 represses it. Also, XAL2 upregulates *SOC1*, *LFY, AP1*, and *TFL1* (Pérez-Ruiz et al., 2015; Figure 1A). All these regulatory interactions were documented under a LD photoperiod, and they were observed when the entire seedling was collected. But the strongest *XAL2* mutants phenotypes were observed under SD photoperiod (Pérez-Ruiz et al., 2015). Since flowering transition relies on developmental and physiological signals under SD conditions (Wang et al., 2009; Quiroz et al., 2021), we searched for the role of XAL2 in flowering regulation in response to aging or GA_3_ addition.

*XAL2* mRNA levels increase in aerial tissues during plant development, peaking around 42 days after sowing (das), and GA_3_ treatment accelerated *XAL2* accumulation when they growth in short-day (Figure 1B). Besides, *XAL2* is induced by GA_3_ in shoot apices as soon as 26 das, compared to non-treated plants, and it is downregulated in a DELLA gain of function mutant; the *gibberellic acid insensitive 1* (*gai-1*; Koornneef et al., 1985), and the *sleepy1-11* (*sly1-11*) mutant affected in GA signal transduction (Eriksson et al., 2006), indicating that *XAL2* is positively regulated by GA (Figure 1C). To investigate the role of XAL2 in the regulation of genes involved in flowering transition in response to GA_3_, we treated wild-type and *xal2* plants with this hormone and collected shoot apices at 26 and 36 das to evaluate early and late developmental events. Other than *XAL2*, only *SOC1* was induced earlier (at 26 das) by GA_3_ (Figure 1D and Supplementary Figure 1). Interestingly, from eight genes analyzed at both times (Figure 1D and Supplementary Figure 1), XAL2 mediates the upregulation of *SOC1*, *LFY*, and *AP1* in response to GA_3_ (Figure 1D). While GA_3_ upregulates *SPL9, SPL15*, *FD*, and *FRUITFULL* (*FUL*) genes independently of *XAL2* (Supplementary Figure 1). We also found that *XAL2* transcript accumulation was reduced in the *spl9-4* and *spl15-1* mutants (Wu et al., 2009) under SD conditions, but not in the *spl3-1* background (Figure 1E), suggesting that SPL9 and SPL15 mediate *XAL2* expression in response to aging.

Thus, *XAL2* is induced early in development probably by SPL9 and SPL15, and in response to GA signaling; but most importantly, *XAL2* partly mediates the response of *SOC1*, *LFY*, and *AP1* to GA_3_. These results further document the various roles of XAL2 during plant development. Though, the question remains: if XAL2 induces those genes similarly in SD and LD, why the *xal2* mutant has a more severe flowering transition phenotype under SD? Therefore, we were interested in analyzing its role in the context of the GRN.

### The flowering transition gene regulatory network integrates signals from endogenous developmental processes, photoperiod and vernalization

For a GRN system-level understanding of XAL2 function in the flowering transition, we integrated the novel results (Figure 1) and previous experimental data into a flowering transition-GRN (FT-GRN) model (Figure 1A and Supplementary Table 2). Based on *XAL2* regulatory interactions we incorporated endogenous aging and GA signals, essential for the flowering response under SD conditions, and the vernalization response which previous models did not consider. Therefore, we aim to resolve a new FT-GRN that could respond to different environmental and internal signals.

**FIGURE 2:**
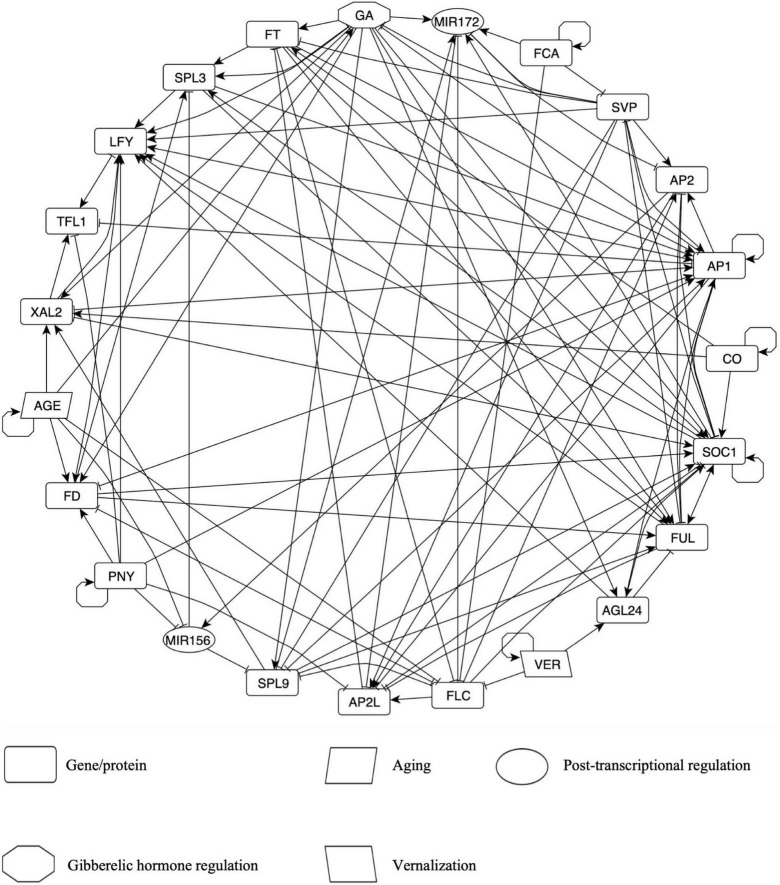
The Flowering Transition Gene Regulatory Network (FT-GRN) in *A. thaliana*, which integrates photoperiod, vernalization, and endogenous signals, is highly connected. Edges represent either positive (arrow) or negative (T-shape line) regulatory interactions. All interactions are based on hand-curated experimental data. Nodes in clockwise order are: GA; MIR172; FCA; SVP; AP2; AP1 includes *CAL* and *AP1*; CO; SOC1; FUL; AGL24; VER, vernalization; FLC; AP2L node includes *TOE1-3, SMZ*, and *SNZ*; SPL9 node includes *SPL9* and *SPL15*; MIR156; PNY node includes *POUND-FOOLISH* (*PNF*) and *PENNYWISE* (*PNY*); FD node includes *FD PARALOG (FDP)* and *FD*; AGE; XAL2; TFL1; LFY; SPL3 node includes *SPL3, SPL4*, and *SPL5*; FT node includes *TWIN SISTER OF FT* (*TFS*) and *FT.* Also see Supplementary Table 2 for details.

The FT-GRN presented here (Figure 2) was derived from a hand-curated literature search reported on the molecular regulators of flowering transition in *A. thaliana*. It integrates regulatory interactions from 146 publications and 45 genes (Supplementary Tables 2, 3), including the novel *XAL2* data shown here (Figure 1). The network nodes represent genes and gene products such as microRNAs or proteins (mainly transcription factors). It also incorporates GA hormone signaling (GA), and processes such as aging (AGE) and vernalization (VER). Genes were selected according to their relevance in the plant’s flowering response to external and internal cues, and functionally redundant genes were collapsed into a single node of the network (Supplementary Table 2).

The FT-GRN has five input nodes: CO, VER, FCA, AGE, and PNY. CO was selected as the input for the simulation of a LD photoperiod because flowering response relies on this protein, although there are many circadian cycle proteins upstream of CO (Suárez-López et al., 2001; Valverde et al., 2004). Vernalization (VER) corresponds to a prolonged period of cold that resembles winter and it is important for *FLC* epigenetic silencing (Sheldon et al., 2002; Whittaker and Dean, 2017), but also in the upregulation of *AGAMOUS-LIKE 24* (*AGL24*) independently of *FLC* inhibition by mechanisms still unknown (Michaels et al., 2003). FCA is a RNA binding protein that acts within a larger protein complex in *FLC* epigenetic silencing in response to aging, independently of photoperiod and vernalization, thus it has been considered as an autonomous pathway of flowering transition (Macknight et al., 1997; Wu et al., 2020). FCA is also involved in the post-transcriptional regulation of miR172 and *SVP* depending on ambient temperature (Lee et al., 2007; Jung et al., 2012). Details about *FCA* transcriptional regulation are unknown, but it regulates itself at the post-transcriptional level to achieve correct levels of its active form (Quesada et al., 2003). The AGE node represents a growth period after some molecules are depleted, accumulated, or become active/inactive. For example, GA levels (Eriksson et al., 2006), *FD* (Abe et al., 2005; Klepikova et al., 2015) and *XAL2* mRNAs (Figure 1) increase as the plant grows. On the contrary, miR156 levels decrease during development (Wang et al., 2009; Jung et al., 2011), like *FLC* (Searle et al., 2006; Klepikova et al., 2015). *PNF* and *PNY* (both included in the PNY node) were included because they are essential in flower meristem formation (Smith et al., 2004; Kanrar et al., 2008), although they have not been considered classical flowering genes. *PNY* regulation is unknown, and thus we kept it as an input in the FT-GRN. Therefore, the model inputs simulate the response to multiple signals from both the environment and endogenous cues.

The FT-GRN also incorporates the *FT* and *TSF* genes (both in the FT node), which are induced by LD photoperiod in the leaves, and then, the protein is transported by the phloem to the SAM (Wigge et al., 2005; Corbesier et al., 2007). FT/TSF and TFL1 compete for the binding of FD and FDP, resulting in the promotion or repression of flowering transition, respectively (Abe et al., 2005; Zhu et al., 2020). The GA node represents high levels of this hormone and the concomitant DELLA proteins degradation (Davière and Achard, 2013). We kept separated *SPL9/15* (in the *SPL9* node) and *SPL3/4/5* (in the *SPL3* node) because they form different clades with unique functions in plant development (Xu et al., 2016). Similarly, *AP2* and *AP2-*like genes were separated into two nodes because *AP2* is related to floral development and *AP2*-like to flowering repression (Mathieu et al., 2009; Yant et al., 2010), although they also shared common functions. The FT-GRN includes MADS-box transcription factors such as SVP and FLC that function as flowering repressors, and others that promote flowering: SOC1, FUL, AGL24, and XAL2. Finally, this FT-GRN incorporates the flower meristem identity genes: *LFY* and *AP1* (together with *CAL* regulation); and the inflorescence meristem identity gene *TFL1* (Ratcliffe et al., 1999).

The first result from this network is that most of the nodes are highly connected among themselves (Figure 2). The FT-GRN has 23 nodes and 121 edges (Figure 2 and Supplementary Table 3) and a directed net density of 0.24 that measures the proportion of edges present within all possibilities in the directed network. In fact, the whole network shows an average of 10.5 regulatory interactions per node (average degree). The analysis of the FT-GRN topology shows that the FT-GRN has a high global transitivity of 0.52, which measures the probability that the adjacent nodes of a node are connected. The network reciprocity is 0.33; this number represents the proportion of reciprocated ties in a directed network; so, there is a relatively high proportion of direct feedback loops in the FT-GRN. Therefore, the FT-GRN is highly connected (density and average degree), topologically redundant (transitivity), and has a high number of feedback loops (reciprocity).

As expected, despite the high connectivity, some nodes are more connected than others (Supplementary Figure 2). The nodes with the highest degree (total number of edges) are SOC1 and AP1. Also, *FUL, LFY, AP1*, and *SOC1* showed the highest indegree values, which measures the number of regulators of each gene (Supplementary Figure 2). It is interesting that *FUL* has an indegree closer to *SOC1*, which is considered an integrator of the flowering pathways, and surprisingly, the number of *FT* regulators is like the number of regulators for other nodes in the FT-GRN. Additionally, the number of genes regulated by a particular node in the network, indicated by the outdegree, showed that *SOC1, SVP*, GA, and *AP1* have the highest values (Supplementary Figure 2), accordingly with their importance as regulators in the flowering transition network.

Contrary to what would be expected if independent or parallel genetic pathways converged to a few integrators to induce flowering transition, we found that all nodes are highly interconnected among themselves. Therefore, the so-called integrators were not identified in the network based on purely topological information. Even if the topology of a network constrains its *dynamics*, the topological properties of the network such as the node’s degree are not enough to understand the function of specific components whiting the whole system, the specific *dynamic* details captured in the node’s logic rules which are different for example in *FT* and *SPL9*, can explain why even if they have the same degree, they have different roles in the *dynamics* of the system. One way to explore the node’s function in the context of the system-level *dynamic* properties of the model is by perturbing individual components (see section “Differentiation and Plasticity Are Emergent Properties of the FT-GRN Signal Sensing and Integration”).

### The flowering transition gene regulatory network Boolean model recovers vegetative and reproductive phase transitions of the shoot apical meristem

**FIGURE 3:**
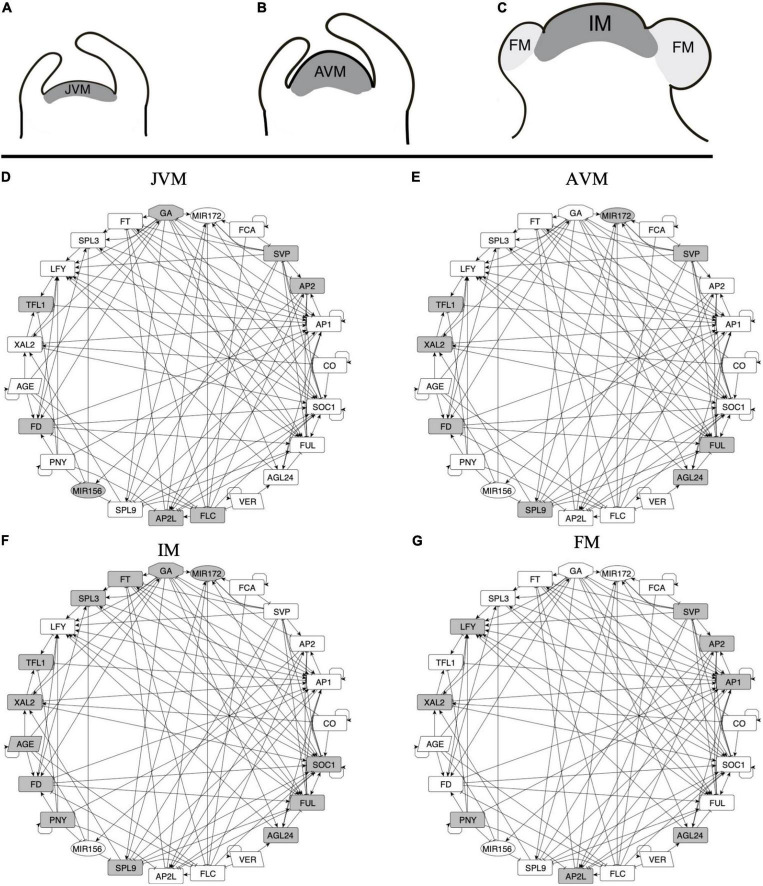
A multi-stable dynamic GRN model underlies the different expression patterns observed during SAM development and FM induction. **(A–C)** Three stages of the SAM can be distinguished during development after germination: the juvenile vegetative meristem (JVM), the adult vegetative meristem (AVM), and the inflorescence meristem (IM). During the vegetative phase of development, the SAM produces leaves, while at the reproductive state, the floral meristems (FM) are formed at the flanks of the IM. **(D–G)** A *dynamic*, multi-stable gene regulatory network underlies the development of the different types of meristems. Nodes in gray are the ones expected to be express in each meristem stage. Positive (arrows) and negative (T-shape lines) interactions are shown.

We hypothesized that a multi-stable FT-GRN module underlies the meristem identity transitions in the development from the vegetative to the reproductive state (Figure 3). In this study, we have integrated many of the molecular components that have been characterized for flowering transition in response to different environmental and internal signals, including genes like the *SPL* transcription factor family that have been shown to participate in developmental transitions and their negative regulation by miR156 (Huijser and Schmid, 2011). During aging, miR156 is downregulated with the concomitant increase of *SPLs* and miR172 expression (Wu and Poethig, 2006; Wang et al., 2009; Wu et al., 2009). The latter represses *AP2* and *AP2*-like TF’s family that act as repressors of flowering transition (Mathieu et al., 2009; Yant et al., 2010). The miR156-SPLs-miR172 module is also important for vegetative transition from juvenile to adult plants (Wu et al., 2009).

We defined the upregulation of *SOC1* (Samach et al., 2000; Immink et al., 2012) and *TFL1* expression (Liu et al., 2013; Baumann et al., 2015) as markers of VM to the IM transition, while the FM identity acquisition was defined by the expression of *LFY* and *AP1* with the concomitant repression of *TFL1* (Shannon and Meeks-Wagner, 1993; Liljegren et al., 1999; Ratcliffe et al., 1999).

The FT-GRN *dynamic* behavior was formalized with a Boolean model (Supplementary Table 4) that captures the regulatory logic between the genes necessary and sufficient for SAM phase transitions and flowering. First, we simulated the *dynamics* of a wild-type FT-GRN by an exhaustive *dynamic* exploration, initializing with all 2^23^ possible states. Despite the immense size of the state space of the FT-GRN model, both, synchronous and asynchronous updating schemes gave the same 32 fixed-point attractors, and zero cyclic attractors (see section “Materials and Methods” and Supplementary Figure 3). This result indicates that the non-linear interactions integrated into the model, based on available experimental data, effectively canalize a large and complex *dynamic* system into a relatively small set of attractors, demonstrating the system’s robustness to initial conditions.

**FIGURE 4:**
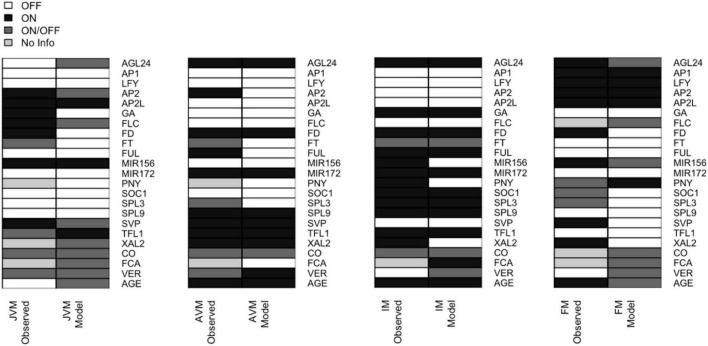
The expression patterns in the attractors recovered by the FT-GRN model are like the ones observed during development. The 32 attractors recovered by the model can be classified into four phenotypes (JVM, AVM, IM, and FM). The gene expression patterns observed *in planta* (left) are compared to the model predicted gene expression patterns (right). The node state can be expressed/active (ON, black) or not expressed/inactive (OFF, white); or can be in either state ON or OFF (dark gray); the unknown expression patterns were label as “No Info” (pale gray). For individual attractors’ information, see Supplementary Figure 3, and for references of gene expression patterns *in planta* consult Supplementary Table 5.

We recovered attractors with similar expression patterns as those observed during SAM phases of development (Supplementary Table 5). Thus, the attractors were classified into the different meristem types: JVM (10 attractors), AVM (2 attractors), IM (4 attractors), and FM (16 attractors). There is more than one attractor per meristem type depending on the initialized input states, thus, we use the term meristem phenotype to distinguish them from the individual attractors obtained. For instance, the model recovers two attractors with the AVM phenotype that differ in the state (on or off) of the CO node, which simulates a LD or SD photoperiod, respectively. The consensus states for the attractor’s phenotypes are shown in Figure 4.

Based on the analyses of the attractors recovered by the model, the conditions driving the JVM to the AVM state are the plant aging, or the acceleration of vegetative development when plants are vernalized. The model predicts that vernalization has an impact on the juvenile-adult phase change because it downregulates *FLC*, and FLC induces *AP2* and *AP2*-like genes and represses *SPL9* and *MIR172* (Deng et al., 2011; Mateos et al., 2015). As expected, downregulation of the flowering repressors, *FLC* and *SVP*, is necessary for the AVM to become an IM. Likewise, the formation of the FM in the model requires *LFY* and *AP1* positive feedback and the presence of *PNY* to block the expression of *TFL1*, driving the system to the floral state. Therefore, the FT-GRN proposed here constitutes a multi-stable, *dynamical* system’s mechanism that sustain previous observations underlying SAM phase transitions.

Some expression patterns obtained from the model attractors differed from the observed profiles *in planta* (Figure 4 and Supplementary Table 5). Such discrepancies can be interpreted in terms of the discrete nature of the Boolean model that is limited to qualitative but not quantitative changes in expression or activity. For example, *FD* is expressed in the JVM, and its expression rises in the AVM and IM (Abe et al., 2005; Wigge et al., 2005); the model does not account for the low expression levels of *FD* at early stages, but it recovers that *FD* is upregulated at the SAM in later stages of development. Besides, *FD* mRNA has been detected in the FM (Abe et al., 2005); but it is negatively regulated by AP1 (Kaufmann et al., 2010b; Pajoro et al., 2014b). Therefore, the model predicts that this gene is turned off in the FM attractors.

Likewise, there is evidence suggesting that SOC1 and AP1 downregulate *XAL2* (Pérez-Ruiz et al., 2015). In the model, these regulations result in the complete repression of *XAL2*. Also, RNA *in situ* hybridization of miR156 shows it at the shoot apex during the plant life-cycle, although its levels decline as the plant ages (Wang et al., 2008, 2009). In the Boolean model miR156 is present in the JVM and absent in the AVM and IM. Basal GA levels are present at the SAM throughout the life span (Eriksson et al., 2006), but they increase in the mature stages of development. In the Boolean model, GA is absent in the vegetative attractors and present in the IM (Figure 4). This can be interpreted as increased GA levels during the flowering transition, rather than their absence in the JVM.

Overall, the model recovered the great majority of documented gene expression patterns during *A. thaliana* developmental stages.

### Getting an inside in the gene expression patterns observed in the shoot and leaves cell-types during the vegetative stage

Zhang et al. (2021) recently published a study in which they used single-cell RNA sequencing (scRNA-seq) to distinguish seven cell types and subgroups from 23 cell clusters based on genes specific expressions that distinguish each cell type in the shoot and leaves (Zhang et al., 2021). This information can be visualized in two-dimensional maps using the uniform manifold approximation and projection (UMAP), and we downloaded those images from their platform http://wanglab.sippe.ac.cn/shootatlas/. Sixteen genes of our network are expressed in different cell types, while *FT* and *AP1* are absent, confirming that the shoots and leaves are at the vegetative stage. Additionally, there was no expression of miR156 and miR172 in the single-cell data, probably due to their instability.

As seen in Supplementary Figure 4, even when some genes are expressed in the same cell type, they have apparent differences in the clusters in which they are identified. For example, *SOC1* is densely expressed in cells that give rise to mesophyll cells (MC), and a group of undefined cells (UC), while *AGL24* is expressed only in one cluster of the MC. Conversely, *AGL24* has a broader distribution in cells that will give rise to vascular cells (VC), proliferating cells (PC) and is highly expressed in shoot meristematic cells (SMC) and epidermal cells (EC) in contrast to *SOC1*. Similarly, *SVP* is expressed in all cell types, whereas *FLC* is restricted to five of them (Supplementary Table 6).

*SPL3* and *SPL9* are expressed differentially. While *SPL3* mRNA accumulates in EC, VC, and companion cells (CC), *SPL9* has higher expression in the MC and different clusters of the EC-type than *SPL3* (Supplementary Figure 4), supporting the idea that they have at least partial independent roles during the vegetative phase of development.

Another example that caught our attention was the expression of *FD*. It is known that TFL1 binds FD in the absence of FT (Zhu et al., 2020). Both genes expression overlaps in the SMC, similarly to the inflorescence meristem. However, *FD* is also expressed in EC, PC, CC, and a different cluster of VC (Supplementary Figure 4). This opens the possibility that FD may act independently of TFL1 or binds other proteins. The fact that *TFL1* was detected in the VC made us suspect that it could be mistaken with other members of the *FT* family; thus, we analyzed the expression in a root single-cell assay http://wanglab.sippe.ac.cn/rootatlas/ (Zhang et al., 2019). *TFL1* is also expressed in root VC (Supplementary Table 6), and *FT* was not found, suggesting that *TFL1* mRNA might be present in the vasculature of vegetative organs. Moreover, scRNA-seq from the root, showed that *AGL24* is almost undetectable with scarce presence in lateral roots and root hair cells, while *SOC1* is highly expressed in different cell types. Further experimental functional data are now required of each SAM stage to be able to recover additional interactions and cell-specific attractors.

### The flowering transition gene regulatory network is robust

Robust GRNs should buffer noisy signals and stochastic biochemical processes while preserving cell phenotypes (Kitano, 2004). The FT-GRN model’s functional robustness was assessed by the invariance of attractors in response to perturbations in the logical rules. First, we tested the robustness of attractors to perturbations in the Boolean functions; afterward, we compared the state transition robustness of the model to random models with the same topological properties; and finally, we approximated the Boolean model to a continuous model to test if the attractors were independent of the modeling framework and the kinetic parameters of the functions.

**FIGURE 5:**
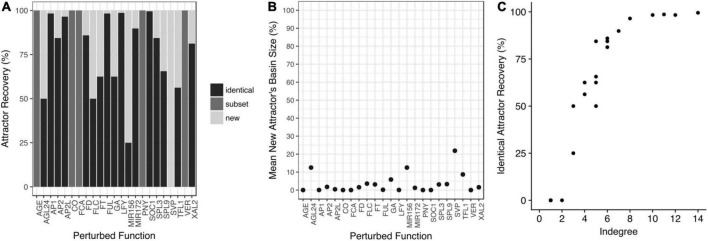
The attractors are robust to perturbations of the Boolean functions. **(A)** Copies of the model were constructed with one-bit random perturbations in each node’s truth table. The attractors recovered by each perturbed model were compared with those obtained with the original model. In some cases, the perturbed models recovered identical attractors to the original model (identical); or they recovered a subset of the original attractors (subset), or both, original and new attractors appeared in the perturbed model (new). **(B)** The mean basin size of the new attractors is small. **(C)** The percentage of identical attractors recovery (robustness) increases with the indegree of the perturbed node.

For each node, copies of the model were constructed with one-bit random systematic perturbations to the truth tables of the Boolean functions. The attractors recovered by each perturbed model were compared with the attractors of the unperturbed model (Figure 5A). The perturbed networks either recovered identical attractors to the original model, a subset of the original attractors, or both original and novel attractors. Only when the input nodes were perturbed, fewer attractors were recovered compared to the original FT-GRN (Figure 5A). Furthermore, the mean basin size of the new attractors in the perturbed models were relatively small compared to the original ones (Figure 5B).

As expected, the perturbations that recovered identical attractors, were the ones that implied nodes with the highest indegree (Figure 5C) and higher redundancy in their regulation. Finally, we tested if some attractors were susceptible to be lost by random perturbations to the Boolean functions and we found that the 32 attractors were maintained with the same frequency when the network was randomly perturbed (Supplementary Figure 5). Thus, the functional robustness analysis revealed that the FT-GRN attractors are robust to perturbations in the Boolean functions (Figure 5).

A biological network is assumed to be robust to small perturbations (Kitano, 2004). We expected attractors in real networks to be less susceptible to noise than attractors in randomly generated networks, since biological processes are relatively stable and emerge even in the face of ever-changing conditions prone to stochasticity. The functional robustness was compared between the FT-GRN model and randomly generated models with the same topological properties. As expected, the FT-GRN model was considerably more robust than random models (Supplementary Figure 6). Therefore, robustness of the FT-GRN is not related only to its topology, but to the *dynamics* of its gene regulatory interactions.

The Boolean model was then transformed to a continuous system of ODE to test whether the mathematical formalism used was determinant of the attractors obtained. This was not the case, as the continuous FT-GRN model recovered the same steady states as the Boolean model (Supplementary Figure 3). The parameters analysis of the FT-GRN continuous model showed the attractors were stable to a range of values in the kinetic parameters. This result indicates that the attractors recovered in the original FT-GRN Boolean model are not an artifact due to the discrete nature of the Boolean model. Instead, such attractors emerge from the overall network topology and logical structure, independently of the kinetic parameters. Besides, it supports the thesis that Boolean models can recover the complexity of biological regulatory mechanisms and thus, they are powerful tools to unveil how genotypes map into phenotypes.

To summarize the results obtained from the robustness analyses, the attractors recovered by the FT-GRN model are stable to discrete perturbations of the Boolean functions and represent the expression patterns of the SAM and FM in the development of *A. thaliana*. Additionally, the robustness of the FT-GRN is not a causality related to the topological properties of the network, but a consequence of the biological regulatory mechanisms involved. Furthermore, the specific meristem configurations are emergent properties of the logic behind the regulatory interactions of the FT-GRN, and they are independent of kinetic parameters.

### The flowering transition gene regulatory network model was validated by comparing simulated mutant phenotypes with actual *A. thaliana* mutant data

**FIGURE 6:**
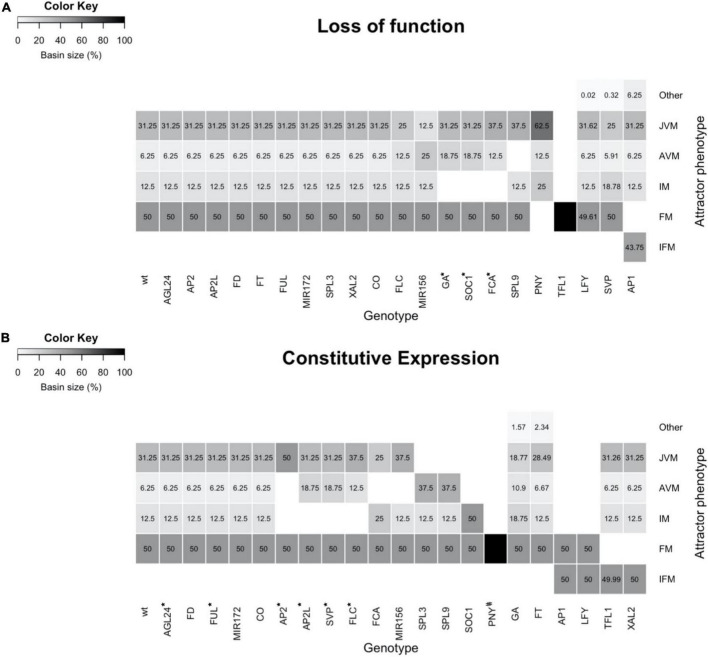
The mutant’s simulations with the FT-GRN Boolean model yield attractors like the observed mutant’s phenotypes. **(A)** Simulation of single loss of function, or **(B)** constitutive expression mutants. The basin size of each meristem state (phenotype) in relation to all state space was plotted as a heatmap. Different types of meristems were specified as JVM, AVM, IM, FM, a chimeric inflorescence-floral meristem (IFM), and other chimeric cyclic attractors (Other). Most simulations recovered the phenotypes described in the literature. However, some mutants’ phenotypes were partially recovered (*), and some mutants have not been described yet (#).

For a further validation of the FT-GRN model, we tested its ability to recover the observed mutant plant phenotypes compared to those observed in wild-type (WT) plants. Single loss-of-function (LOF) mutants and constitutive expression (CE) lines were simulated by maintaining each node state at 0 or 1, respectively. We assessed if novel phenotypes were recovered compared to the WT plants simulation (Figure 6). For this purpose, the cumulative basin of attraction size for a particular phenotype, corresponding to the sum of the state space that converge into attractors with the same meristem type in relation to all the state space, was compared between the mutant and the WT. Finally, the *in silico* mutant predictions were compared to the experimental data available for each mutant phenotype to assess if such data was recovered by the model (Figure 6 and Supplementary Table 7).

Most of the LOF simulations showed similar attractor’s phenotypes as the WT (Figure 6A), consistent with most of the single LOF mutant plants having mild phenotypes (Supplementary Table 7). However, a few LOF mutants completely lost some of the WT attractors, also changing the basin size of the remaining attractors in the state space. This was the case for low levels of GA, *soc1* and *fca* mutant simulations, in which the IM was lost, and the basin size of AVM attractors was increased compared to the WT. The same happened to the JVM basin of the *fca* mutant. In agreement with this, the *ga1-3* mutant is late flowering in a LD and in a SD, flowering fails completely (Wilson et al., 1992). *soc1* and *fca* plants are very late flowering (Koornneef et al., 1991; Onouchi et al., 2000), and the latter produce more vegetative juvenile and adult leaves than WT plants (Telfer et al., 1997).

Of interest, the *spl9/spl15* simulation showed that plants remain at the JVM state and transit to the IM without developing an AVM, in agreement, the *spl9 spl15* double mutant has a prolonged juvenile phase (Schwarz et al., 2008; Xu et al., 2016). The PNY LOF simulation does not show the FM attractors. Correspondingly, the *pny pnf* double mutant does not develop flowers when the flowering time genes are upregulated (Smith et al., 2004). Conversely, nullifying TFL1 in the model predicts only FM attractors, losing the vegetative and IM states, likewise the *tfl1* plants flower very early, and their SAM is rapidly transformed into a terminal flower (Shannon and Meeks-Wagner, 1991).

By observing some puzzling results when we compared the mutant simulations with the documented mutant phenotypes, the model revealed a connection between the vegetative phase change and its effect in the flowering transition. In particular, the simulation of *FLC* or miR156 LOF mutants recovered a smaller JVM basin compared to the one observed in the WT, but they also showed an increased AVM basin compared to the WT. These basin changes may be interpreted biologically as a faster vegetative phase change. It has been reported that *35S::MIM156* lines that show reduced function of miR156, only produce rosette leaves with adult morphology (Franco-Zorrilla et al., 2007; Xu et al., 2016), while *flc* mutation promotes adult vegetative traits both dependently and independently of the flowering time (Willmann and Poethig, 2011). Nonetheless, LOF of FLC or plants with reduced miR156 activity, also have an early flowering phenotype, that was not recovered by the simulated LOF mutants due to the presence of the flowering repressor SVP, which is independent of miR156 and FLC levels, preventing early flowering transition in the model. These results suggest unknown negative regulators of *SVP*, which would be necessary for the early flowering phenotype in the model when *FLC* or *MIR156* are mutated. The double LOF mutants *svp flc* or *miR156 svp* simulations had a larger IM basin size compared to the WT (Supplementary Figure 7). *svp flc* and *svp miR156* had an IM basin size of 25 and 18.8% of the state space, respectively, while the IM basin size was 12.5% in the WT, this predicts early flowering phenotypes and shows that *SVP* has a redundant role as a flowering repressor.

Furthermore, the *svp* simulation resulted in reduced AVM and JVM basins, a larger IM basin, and a cyclic attractor with an AVM/IM phenotype (Figure 6A). Accordingly, the *svp* mutant plants are early flowering, and they produce fewer rosette and cauline leaves than wild-type plants (Hartmann et al., 2000). The predicted AVM/IM phenotype in *svp*, suggest that this gene is relevant for maintaining the vegetative stage. The role of *SVP* during the vegetative phase beyond its role as a flowering repressor needs further research.

*lfy* mutant plants have partial transformations of flowers into inflorescence shoots, and secondary flowers generally arise from the axils of the outer floral organs; these plants also have additional secondary inflorescence shoots subtended by cauline leaves (Weigel et al., 1992). In agreement, it is remarkable that the *lfy* simulation recovered novel cyclic attractors with a mixed FM/IM phenotype at the expense of a slight reduction in the basin of FM attractors under SD conditions. Besides, the AP1 LOF simulation does not present the FM attractors while it recovered chimeric inflorescence-floral meristem (IFM) attractors as well as additional JVM/FM/IFM cyclic attractors. The novel attractors of the *AP1* LOF simulation are consistent with the observed phenotypes in real *ap1-1* mutant flowers, including the formation of bract-like first whorled organs and floral buds in their axils (Irish and Sussex, 1990).

In contrast to LOF mild phenotypes, simulations of constitutive expression (CE) lines predicted more drastic alterations, including loss of some types of meristems and novel meristem phenotypes (Figure 6B). CE of the flowering repressors *FLC, SVP, AP2*, and *AP2-*like genes, resulted in the disappearance of the IM attractors and a larger basin of the vegetative meristem ones. The predictions agree with phenotypes observed in the overexpressor lines of these genes, which are late flowering and, in some cases, remain vegetative throughout their complete life cycle (Supplementary Table 7; Michaels and Amasino, 1999; Chen, 2004; Masiero, 2004; Lee et al., 2007; Mathieu et al., 2009; Yant et al., 2010). In the *FCA* CE simulation, the JVM basin was reduced compared to the WT, and the AVM state disappeared, while the IM basin of attraction increased. The *FCA* overexpressor line is early flowering, although it is not as drastic as the simulation, probably because there are different ratios in the abundance of *FCA* alternative transcripts, adding another level of regulation that is not considered in the model (Macknight et al., 1997). Like the FCA CE simulation, the AVM attractors in the miR156 CE simulation disappeared, but in contrast to CE of FCA, a larger JVM basin was obtained in the miR156 CE line than in the WT. This result agrees with the prolonged juvenile vegetative phase phenotype observed in miR156 overexpressor plants (Wu and Poethig, 2006; Schwarz et al., 2008).

Conversely, the JVM attractors disappeared in the *SPL3* and *SPL9* CE simulations, and instead, a larger AVM basin was predicted compared to the WT. Indeed, *SPL3, SPL4, SPL5, SPL9*, and *SPL15* overexpressor plants transit from their juvenile phase to further stages faster, and adult traits appear earlier than in WT plants (Cardon et al., 1997; Wang et al., 2008; Yamaguchi et al., 2009). In *SOC1* CE simulation, the vegetative state of the SAM is lost, which correlates with the observed effect of *SOC1* overexpression, causing very early flowering in both SD and LD (Borner et al., 2000). In addition, the model predicts that the PNY CE forms only FM attractors; however, this overexpression line has not yet been described in the literature.

In some CE simulations, new phenotypes were predicted besides the ones observed in WT plants. Increased GA response simulation had larger AVM and IM basins and a smaller JVM basin compared to the WT, but it also recovered additional cyclic JVM/AVM attractors. Mutants in *SPINDLY* (*SPY*), a GA signaling repressor, resemble wild-type plants that have been repeatedly sprayed with GA_3_; *spy* plants and GA_3_-treated WT plants are early flowering and they also produce adult vegetative leaves earlier (Jacobsen and Olszewski, 1993; Telfer et al., 1997). Similarly, simulation of FT CE also predicted a smaller JVM basin and recovered additional cyclic attractors with a JVM/AVM phenotype. Indeed *35S::FT* plants are early flowering (Kardailsky et al., 1999; Kobayashi et al., 1999; Hanzawa et al., 2005), but there is no information related to the morphological traits like the presence of abaxial trichomes distinctive of mature leaves *versus* juvenile leaves in these transgenic lines. Finally, other CE mutant simulations had novel phenotypes at the expense of losing others. For example, *AP1* and *LFY* CE simulations, predict that the SAM converts into the FM or a chimeric IFM meristem. Actually, *35S::AP1* plants are early flowering, and the SAM becomes a floral meristem that produces a terminal flower (Mandel and Yanofsky, 1995), besides, *35S::LFY* plants are also early flowering and they develop solitary flowers instead of secondary shoots (Weigel and Nilsson, 1995). On the contrary, the FM disappeared in *TFL1* and *XAL2* CE simulation, and a chimeric IFM meristem is predicted, as has been reported in the literature for these mutant lines (Ratcliffe et al., 1998; Pérez-Ruiz et al., 2015).

Although most of the LOF and CE model predictions were congruent with mutant plants described in the literature (Figure 6), some discrepancies were found. The model did not recover the chimeric IFM observed in the overexpression lines of *AGL24*/*SVP* and the *agl20-101D* (Yu et al., 2004; Liu et al., 2007), which are similar to the *35S:XAL2* phenotype (Pérez-Ruiz et al., 2015). Our previous discrete model, had different conditional rules for loss of function mutants, the WT expression, and gene overexpression lines (Pérez-Ruiz et al., 2015). In order to translate the discrete multi-valued *dynamics* into a Boolean model, we followed the methodology in Chaos et al. (2006). Therefore, the conditional induction of *TFL1* in those overexpressors was lost.

We also found that the flowering time phenotypes of *co*, *ft*, *fd*, *agl24*, *ful*, and *xal2* mutants (Supplementary Table 7) were not reflected in changes of the size of the VM or IM basins (Figure 6). This can be explained because the model is not quantitative and as such, it is not meant to give quantitative information of flowering time as other models have done (Jaeger et al., 2013; Wang et al., 2014; Leal Valentim et al., 2015; van Dijk and Molenaar, 2017; Haspolat et al., 2019).

A third group of *in silico* mutants were partially recovered because they lost the IM state which correlates with their late-flowering phenotypes, but they still recovered the FM states, which should be lost. This group includes the simulation of GA insensitive mutants, *SOC1* and *FCA* loss-of-function, and constitutive expression of *AP2, AP2L, SVP*, and *FLC.* The few discrepancies between the model’s predictions and experimental mutant data yielded novel hypotheses that should be tested experimentally.

### XAL2 is an important component of the flowering transition gene regulatory network

**FIGURE 7:**
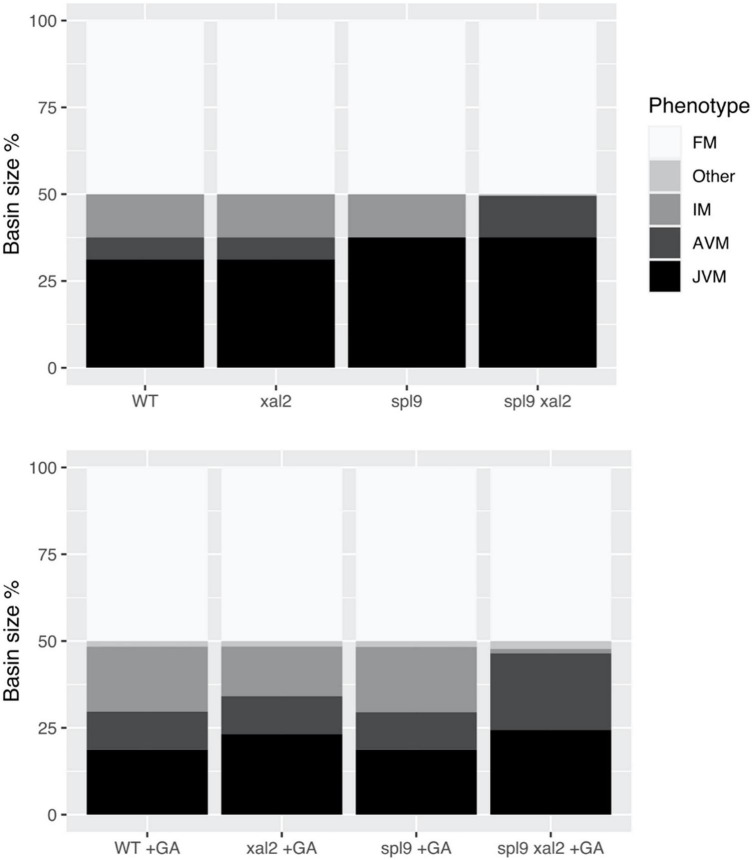
*XAL2* is an important component of the FT-GRN that mediates GA promoting flowering response in SD. We performed mutant simulations in SD with or without GA treatment, fixing the FT-GRN model to simulate SD by keeping the CO input turned off, and GA exogenous treatment was simulated by keeping the GA node turned on. Wild-type, *xal2*, and *spl9* single and double mutants were simulated with (lower panel) or without GA (control, upper panel). For each mutant background under a specific “growth” condition we calculated their attractors, classified them in meristem phenotypes (JVM, AVM, IM, FM, and “Other” which were vegetative cyclic attractors), and plotted their relative basin size as a percentage of all the initial state space.

We further investigated the role of *XAL2* in the context of the FT-GRN. We explored *XAL2* mutant phenotypes simulating SD conditions by keeping CO input turned off in the model and we also analyzed the GA response in this condition (Figure 7). Simulation of wild-type plants treated with GA shrank the JVM basin size and enlarged the AVM and IM state basins compared to non-treated plants in SD, this agrees with the promotion of flowering by GA_3_ treatment in SD. However, simulation of *xal2* mutant treated with GA_3_ showed a smaller basin size of the IM state than the WT treated with GA.

In SD, *A. thaliana* flowering depends on endogenous cues like hormonal and aging signals (Quiroz et al., 2021). The miR156 – SPLs – miR172 – AP2-like module is important for aging developmental changes, and SPL9 and SPL15 positively regulate *XAL2* (Figure 1E). Simulation of *spl9/spl15* in SD lacked the AVM stage, congruent with *spl9 spl15* plants having a longer juvenile phase (Schwarz et al., 2008; Xu et al., 2016). However, simulating *spl9/spl15* treated with GA in SD recovers the AVM and behaves similarly to GA-treated WT plants, suggesting that GA signaling is at least partially independent of SPL9/SPL15. Interestingly, when the double mutant *spl9 xal2* was simulated in SD, the IM state was lost. Moreover, when *spl9 xal2* double mutant was treated with exogenous GA *in silico*, it could not recover the IM basin size and instead had a larger vegetative basin size, showing that the introduction of *xal2* null-function mutant compromised the GA response. The model predictions support the notion that XAL2 is part of the flowering response to GA in SD, but generation of *spl9 xal2* and *spl15 xal2* double mutants and empirical confirmation are required.

### Differentiation and plasticity are emergent properties of the flowering transition gene regulatory network signal sensing and integration

By considering the attractors recovered by the FT-GRN as specific meristem types, we were interested in understanding how the SAM stages are acquired, and how the sequential pattern of such transitions emerges. Furthermore, we wanted to understand how such phase transition pattern is altered under different environmental and developmental cues. First, we searched for the nodes that could reshape the attractors landscape in a continuous model; then, we explored how to induce differentiation by transitory perturbations of the nodes in the Boolean model and finally, we found that the temporal sequence of differentiation observed in *A. thaliana* is a response to input signals propagation throughout the FT-GRN.

The attractors landscape (Waddington, 1957) corresponds to the space of dimension “n” (*n* = the number of nodes in the network), formed by the attractor states at the bottom of the “landscape” valleys, together with all the other states of the system. Such n-dimensional space emerges from the network structure, topology and *dynamics*, and it constrains the transitions among attractors and also underlies generic time-ordered patterns of temporal transitions among cell types that are observed in actual organismal morphogenesis (Villarreal et al., 2012; Davila-Velderrain et al., 2015b). By recovering and analyzing such attractors landscape for the network proposed in this study, we uncovered which nodes of the FT-GRN may be key for phase transitions. Specifically, the attractor landscape of the FT-GRN continuous model was explored by modifying the decay rates of each node (Davila-Velderrain et al., 2015b) and identifying those that were key for the transition from one SAM phase to another one using previously proposed approaches (Alvarez-Buylla et al., 2008; Huang et al., 2009; Villarreal et al., 2012; Davila-Velderrain et al., 2015b).

**FIGURE 8:**
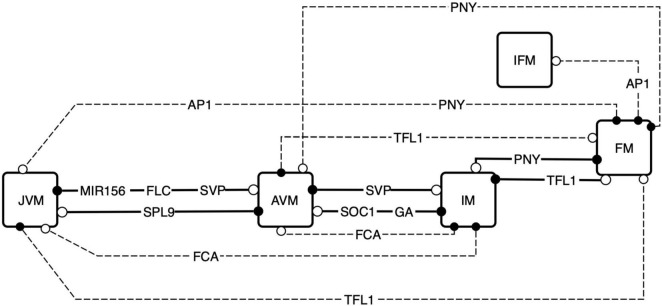
Exploration of the attractors landscape uncovers the regulators that are sufficient for SAM phase transitions. By controlling the decay rates of the FT-GRN components, the attractors landscape is reshaped, and the states of the system can be driven to a contrasting phenotype. The FT-GRN components specified are those which drive changes when they decay faster in a continuous model, this effect is like a conditional gene silencing construct or protein inactivation inducing a phenotype transition. Continuous lines represent model predictions that agree with developmental observations, and discontinuous lines are predictions that have been documented under specific experimental conditions such as gene silencing. Black circles correspond to the initial meristem stage, and the white circles correspond to the final stage after the node was shut down.

*In silico*, it was possible to transit between all meristem types when forcefully maintaining specific nodes shutdown (Figure 8). We found that the vegetative phase transition is controlled by: MIR156, FLC, SVP, SPL9, VER, and AGE nodes in the model, whereas FCA, SOC1, GA, and SVP controlled flowering transition. Shutting-down *TFL1* results in FM fate while silencing *AP1* or *PNY* mediated FM transition to other meristem types. For example, we found the appearance of an additional chimeric inflorescence-floral meristem (IFM) phenotype can be reached by the system when *AP1* is silenced if it starts from the FM state. Not all nodes have the same propensity to modulate differentiation; altering one of ten nodes: MIR156, FLC, SPL9, SVP, SOC1, GA, FCA, PNY, TFL1, and AP1, was sufficient to cause differentiation from one phenotype to another one, whereas the other thirteen did not cause meristem transitions when they were conditionally downregulated *in silico.*

Surprisingly, FT or LFY *in silico* perturbation were not enough to drove the system to differentiation. Furthermore, even FT and SPL9 have the same in and out-degree (Supplementary Figure 2), *in silico* perturbation of SPL9, but not FT, was enough to change the phenotype in the simulation. We searched for components of the FT-GRN driving differentiation when transiently perturbed; the results showed that only input’s perturbations, but not others, were sufficient to drive differentiation (Supplementary Figure 8). This result further supports that the FT-GRN is robust, and more important, that differentiation driven by the FT-GRN occurs in response to signal sensing and integration. The developmental temporal sequence JVM-AVM-IM-FM observed in *A. thaliana* is driven by changes in the inputs AGE, VER, FCA, and PNY, but not by transient perturbations in the CO node. This may seem to be contradictory since *A. thaliana* reproductive development is induced by LD photoperiod, however, considering that it is a facultative flowering plant; it can bloom under SD conditions. The results of the FT-GRN model highlight that the developmental sequence JVM-AVM-IM-FM is robust and independent of photoperiod conditions even if reproductive development is faster under LD than SD. Therefore, developmental transitions are driven by specific signals such as aging (AGE and FCA), vernalization (VER), and spatial cues at the SAM (PNY).

**FIGURE 9:**
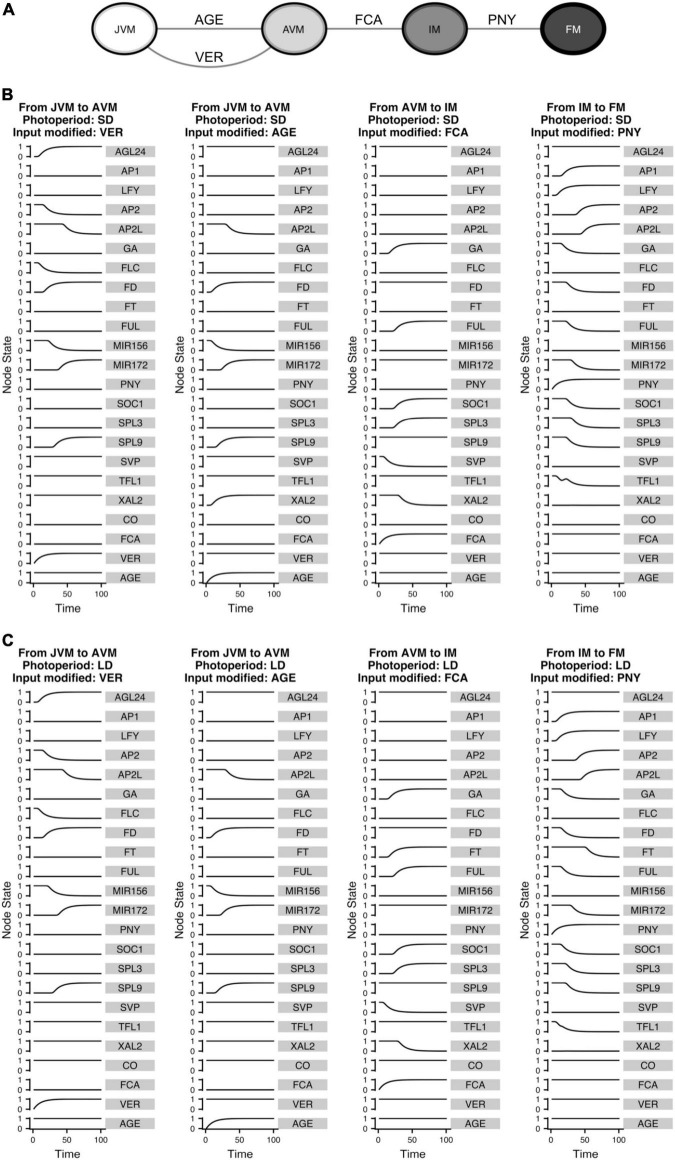
The FT-GRN underlying signal sensing and integration drives the developmental trajectories from the vegetative to the reproductive stage of development. **(A)** Turning “ON” the input nodes can drive the system from one phenotype to another, recovering the temporal sequence of differentiation in response to environmental (VER), endogenous (AGE and FCA), and spatial (PNY) signals. Differentiation simulations for **(B)** SD and **(C)** LD photoperiods, the VER and AGE nodes drive the temporal progression from JVM to AVM; while upregulation of FCA drive the system from the AVM to the IM stage and increasing PNY drives the IM to the FM state.

Reshaping the attractors landscape associated with a GRN *dynamical* model by modulation of gene’s expression helps to understand the role of particular genes in development in the context of the complex GRN involved (Davila-Velderrain et al., 2015b). This approach was applied to the continuous FT-GRN model to assess how the network transduces input signals into developmental transitions (Figure 9). This analysis is like the differentiation trajectories found in the Boolean model (Supplementary Figure 8), but it is possible to visualize the change of each gene over time following the development in response to the input signals.

The inputs that drive the developmental trajectories from JVM to AVM (AGE and VER), AVM to IM (FCA), and IM to FM (PNY) were “turned on” *in silico*. This allowed us to observe how the information was propagated through the network as the relative gene expression levels changed until reaching a new steady state (Figure 9). Interestingly, similar expression trajectories occurred in both SD and LD development simulations for most nodes, except for *FT* which, as expected, was upregulated in LD, but not in SD during flowering transition. Interestingly, *XAL*2 was differentially expressed in response to aging between SD and LD during the vegetative phase transition. Considering that CO or other LD signal regulates *XAL2* independently of *SPL9* (or other component of the aging module), the model predicts that *XAL2* should be expressed in the JVM earlier in LD than in SD growing plants (Figures 9B,C). Another difference between SD and LD was the dynamics of *TFL1* expression to allow the FM fate acquisition. Under SD and LD, the model predicts that *TFL1* is shut down but not in a linear way; the curve of *TFL1* expression during the floral fate acquisition (IM to FM) in LD is smoother than under SD, where a small bump is observed before the subsequent decline (Figures 9B,C). This may imply that an additional dynamical barrier needs to be surpassed under SD conditions to attain FM identity compared to LD. Indeed, it is more likely to observe an incomplete commitment to the FM state under SD than under LD in *A. thaliana* and other plants (Okamuro et al., 1996; Tooke et al., 2005).

The model showed that PNY is an essential component of the network, allowing the system to converge to the floral meristem (Figures 8, 9), even so, there is not much information about its regulation. SOC1 may regulates *PNY*, but the evidence was inconclusive (Immink et al., 2012; Andres et al., 2015). The results obtained in this work led us to hypothesize that XAL2 or SOC1 may regulate this gene. Therefore, we tested this hypothesis and found that *PNY* relative mRNA accumulation in *soc1* and particularly in *xal2* mutants showed lower levels than WT in apices of SD growing plants (Supplementary Figure 9), suggesting that MADS-domain TF regulate this important homeobox. Hence, this FT-GRN is helpful to identify missing interactions when there is insufficient information about some gene’s regulation.

## Discussion

In this work we proposed a multi-stable complex Flowering Transition Gene Regulatory Network (FT-GRN) that is a system-level mechanism underlying SAM phase transitions from vegetative to reproductive states. The FT-GRN is based on three decades of experimental data on previously studied pathways in response to both internal and external cues. The approach presented here enables a formal and *dynamical* means to study how plants respond to and integrate different environmental, genetic, and physiological signals, that trigger reproductive development (Quiroz et al., 2021). This has been possible, thanks to the exhaustive functional, genetic, and molecular studies including tens of components that have been characterized in the model plant *A. thaliana*.

The FT-GRN Boolean model presented here integrates previously characterized pathways, including 45 components (genes, transcripts, proteins, and hormones) and their interactions, based on more than 100 publications and novel data presented here (Figure 1). The reduced 23-node FT-GRN is highly connected, and it retains all functional circuits including 121 edges (Figure 2). Our model recovered the expression profiles of the components involved in the FT-GRN that have been documented in the JVM, AVM, IM, and FM states under different environmental conditions (Figure 4). The FT-GRN was also validated by simulating loss- and gain-of-function mutants, showing that their altered configurations were recovered (Figures 6, 7). Furthermore, the predictive strength of this model was confirmed specifically for *XAL2*, a MADS-box TF, that is implicated in SD and LD flowering promotion (Figures 4, 6, 7). The results suggest that the proposed FT-GRN Boolean model integrates *A. thaliana* necessary and sufficient regulatory interactions in response to different endogenous and environmental signals for SAM developmental vegetative and reproductive phase transitions.

As expected, several analyses indicate that the attractors recovered by the FT-GRN model are functionally robust to perturbations (Figure 5 and Supplementary Figures 5, 6). Furthermore, the approximation of the Boolean model to a continuous system of ODE showed that the mathematical formalism used did not affect the model attractors and *dynamics*. Thus, the proposed FT-GRN behavior and attractors depend on its topology and logic architecture, rather than on the detail of the kinetic functions, as shown for other biological systems (Albert and Othmer, 2003; Li et al., 2004).

The continuous FT-GRN model underlies an attractors landscape that we recovered to explore morphogenetic temporal pathways and to address the critical nodes involved in the transition from one SAM state to another one. As a result, we recovered essential nodes such as MIR156, FLC, SVP, SPL9, SOC1, GA, FCA, PNY, AP1, and TFL1 involved in both the differentiation and plasticity of the system (Figures 8, 9). Interestingly, LFY was not sufficient to drive differentiation and plasticity of the system, even though its relevance in FM initiation. This may be explained because its functional redundancy with AP1 (Weigel et al., 1992; Bowman et al., 1993).

We also showed that the FT-GRN is more robust than random architectures with the same topological properties, emphasizing that system robustness emerges from the integrated biological regulatory *dynamics* based on experimental data, rather than only on the network’s topology (Supplementary Figure 6). Notably, we found that FT and SPL9 have the same out-degree and in-degree (Supplementary Figure 2), but the attractors landscape analysis showed that SPL9 could drive differentiation from one meristem state to another one when perturbed, but this was not the case for FT (Figure 8). Similarly, FCA and CO have the same in and out-degree, but distinct differentiation outcomes are observed when they are “turned on” in the FT-GRN model (Figure 9). It seems that the endogenous flowering signals mask the effect of FT and CO perturbation in our discrete model, pointing to the requirement of endogenous signals in both SD and LD.

### Function of *XAL2* and *SPL* genes in the context of the network

Previous work showed that XAL2 participates in flowering transition, both as a regulator of flowering time and as a regulator of meristem identity genes, particularly as a positive direct transcriptional activator of *TFL1* (Pérez-Ruiz et al., 2015). Here we focused on the role of XAL2 as a flowering promoter under short days because the strongest phenotypes of *xal2* mutants are observed under this growth condition (Pérez-Ruiz et al., 2015), while a previous model had only considered its role for long days. Therefore, we explored *XAL2* regulatory interactions under SD and found that *XAL2* is positively regulated by SPL9 and SPL15 (Figure 1), which are part of the aging module (Quiroz et al., 2021). Results showed that XAL2 is necessary for the GA regulatory response of *SOC1*, *LFY*, and *AP1* under SD conditions (Figure 1). The integration of the FT-GRN model allowed us to simulate different “growth” conditions *in silico* for both wild-type and mutants (Figure 7). The FT-GRN model shows that XAL2 is important for GA flowering promotion response particularly under SD, in agreement with the experimental data shown here. The model also predicts a XAL2 developmental effect in the vegetative phase change since this transition is compromised in the simulated *spl9 xal2* double mutant that can be validated experimentally in the future (Figure 7). Therefore, the prediction suggests that SPL9/SPL15 and XAL2 participate in several phase change transitions of the SAM. In this regard, it is known that SPL9 participates during the vegetative phase transition in *A. thaliana* (Xu et al., 2016).

However, new findings showed that SPL9 is not only controlled by miR156 and GAs, but other endogenous signals also participate. Brassinosteroids (BR), promotes vegetative phase transition through regulating the activity of *SPL9* at transcriptional and post-transcriptional levels. Moreover, the BRASSINAZOLE-RESISTANT 1 (BZR1), a key transcription factor of BR signaling pathway, interacts with SPL9 to cooperatively regulate the expression of downstream genes (Wang et al., 2021). It is interesting, that BZR1 and BRI1-EMS-SUPPRESSOR 1 (BES1), also upregulate *FT* and *SOC1* and repress *DELLAs* and *FLC* (Bao et al., 2019), strongly suggesting BR involvement in SAM’s phase transitions. Further studies can be done on the role of this hormone in *XAL2* regulation and its role in the context of our network.

To our knowledge, this work includes, for the first time, the miR156 – SPLs – miR172 – AP2-like aging module as part of a flowering *dynamic* model, and the inclusion of this module in the FT-GRN was surprisingly informative. With the FT-GRN we could differentiate the juvenile and the adult vegetative states of the SAM and postulate a system-level developmental mechanism to underlie both the vegetative phase change and the transition to a reproductive state. In this sense, the FT-GRN Boolean model showed that regulatory interactions underlying both juvenile to adult vegetative phases transitions, are tightly interlinked to regulatory modules involved in the transition to inflorescence and flowering developmental stages. During the vegetative phase of development, flowering-promoting genes are prevented from being expressed until they are required. The model also suggests that flowering promoting signals accelerate the vegetative phase transition.

The simulations of mutants (Figures 6, 7) often showed that flowering inductive genes also affect the vegetative phase change, while only at maturity they trigger the reproductive phase (Telfer et al., 1997; Willmann and Poethig, 2011). For example, the model suggests that high GA levels or constitutive expression of *FT* may promote the vegetative phase change. There is no information on the juvenile to the adult vegetative transition of extremely fast flowering plants, like *FT, SOC1, XAL2*, or *AGL24* overexpressor lines. However, high levels of gibberellins promote abaxial trichome production in *A. thaliana*, a classic trait that characterizes the adult vegetative phase (Telfer et al., 1997) and, it has been discovered that FT-signaling regulates the transcription of *SWEET10*, a bidirectional sucrose transporter in the leaves (Andrés et al., 2020), that could accelerate vegetative development by mobilizing carbohydrate resources. Hence, our model revealed that flowering signals and genes known to function in the reproductive transition could also participate in the vegetative phase change, and conversely, the vegetative phase change is necessary for reproductive competence.

We also found that PNY is necessary for flower meristem formation in the context of the FT-GRN network (discussed below). *PNY* regulation is almost unknown, although it had been described as a possible target of SOC1 (Immink et al., 2012; Andres et al., 2015). The results obtained in this work led us to hypothesize that XAL2 or SOC1 may regulate this gene. Indeed, we found that *PNY* mRNA accumulation is lower in *xal2* and *soc1* mutants than in WT apices of SD growing plants (Supplementary Figure 9). Therefore, the model has proven to be a helpful framework to integrate available experimental data, detect evidence holes and to postulate novel hypotheses or predictions that could not be proposed without such a systems biology framework.

### The flowering transition gene regulatory network underlies both inflorescence meristem and floral meristems patterning

Previously, an exploration of plasticity by stochastic simulated perturbations of a floral organ specification (FOS) Boolean GRN model (Alvarez-Buylla et al., 2008) suggested that the inflorescence meristem hardly transited into other floral organ basins when low noise levels were used. When larger magnitudes of noise were simulated, the system directly jumped to the attractors corresponding to gene expression configurations of stamens or carpels (Alvarez-Buylla et al., 2008). We postulated that several components and interactions linking SAM states and floral organ attractors could be missing in the FOS model, and this could hamper a more detailed and joint exploration of the attractors landscape. In *A. thaliana*, floral meristems develop at the flanks of the SAM and only some mutations enable the SAM to differentiate into a terminal flower (Shannon and Meeks-Wagner, 1991; Mandel and Yanofsky, 1995; Kardailsky et al., 1999; Kobayashi et al., 1999). On the other hand, the model of Dinh et al. (2017) stressed the importance of spatial cues at the IM to establish the identity of lateral organs through explicit references to this aspect, by the inclusion of an “apex” node and an auxin-maxima node. Certainly, hormones such as auxin (Li et al., 2013), cytokinins (Denay et al., 2017), and BR (Hepworth and Pautot, 2015) have been implicated in FM initiation, formation and separation from the IM. Also, other signals like reactive oxygen species have a role in flowering transition and meristem maturation (Huang et al., 2021). Because FM forms at the flanks of the IM in a specific spatial distribution, coupling GRN *dynamics* with different physicochemical fields including those signaling cues, together with mechano-elastic forces emergent from cellular dynamics and meristem structure could explain FM initiation and separation from the IM.

In our model the PNY node drives the system from the SAM vegetative and inflorescence attractors to the FM attractors (Supplementary Figure 8 and Figure 9). Furthermore, explorations of the attractors landscape (Figure 8) suggest that development of floral meristems requires additional interactions involving PNY. The PNY/PNF-SHOOT MERISTEMLESS (STM) complexes (Cole et al., 2006; Rutjens et al., 2009) regulate maintenance of the SAM and they specify FM emergence in reproductive development by maintaining a boundary between the central and peripheral zones at the SAM and restricting organogenesis to the periphery of the apical meristem (Ung et al., 2011; Khan et al., 2015). The *pny pnf* plants initiate compact shoots that fail to form flowers even though a subset of inflorescence meristem identity genes are expressed in the SAM (Kanrar et al., 2008; Lal et al., 2011; Andres et al., 2015). PNY/PNF-STM are required for induction of the floral identity genes *AP1* and *LFY* by the FT-FD-SPL3/4/5 or by SOC1-AGL24 complexes during inflorescence development (Kanrar et al., 2008; Lal et al., 2011; Smith et al., 2011; Andres et al., 2015). Thus, it is probable that the PNY node, which represents both *PNY* and *PNF* genes in the model, connects the FT-GRN to other regulatory modules that include spatial cues and cellular dynamics at the inflorescence apex. Conversely, there might be feedback mechanisms between the flowering genes and *PNY* as suggested by our experimental data.

### The flowering transition gene regulatory network contributes toward the systems biology understanding of reproductive development

Systems biology approaches are required to provide mechanistic understanding of development (Albert and Thakar, 2014; Davila-Velderrain et al., 2015a). In this work, we have used a GRN Boolean model to address the system-level mechanisms underlying *A. thaliana* reproductive development. Previous models captured important aspects of flowering time under LD conditions, and under the assumption of flowering pathways and integrator genes that transfer information to *AP1* as a quantitative output (Jaeger et al., 2013; Wang et al., 2014; Leal Valentim et al., 2015; van Dijk and Molenaar, 2017; Haspolat et al., 2019). The latter models relied on continuous variables optimized to calculate flowering time, which was modeled as a variable dependent on *AP1* relative expression levels. Therefore, those models have helped us understand important system-level aspects of flowering time such as: the commitment and irreversibility of development, integration of noisy signals, the importance of cooperativity and feedback loops, and prediction of missing interactions (Jaeger et al., 2013; Leal Valentim et al., 2015).

Here, we propose a different modeling framework to previous modeling approaches, that although being qualitative, it integrates experimental data on a larger number of regulatory components and enables formal analyses of the role of environmental cues in flower transition. Even if it does not capture quantitative differences in flowering time, it constitutes a *dynamical*, multi-stable system that integrates previously identified genetic flowering transition pathways to explain SAM phase transitions in both vegetative and reproductive development. Furthermore, the robustness analyses performed showed that the FT-GRN is a robust biological module, and the recovered attractors persist under different initial conditions, even when kinetic functions were altered to produce topological changes.

The model *dynamically* canalizes external and internal signals into the JVM – AVM – IM – FM developmental sequence as it is qualitatively observed in plants (Figure 9). This suggests that the *dynamics* of the underlying GRN establish developmental checkpoints, including the commitment to the reproductive phase. We showed that the same regulatory network explain how flowering transition is induced in response to internal cues modulated by environmental signals. The comparison between SD and LD developmental trajectories suggests that endogenous signals are necessary to overcome flowering repressive signals (Figure 9), even when long-day photoperiod accelerates this transition (Quiroz et al., 2021).

Development emerges from the complex interaction between growth and differentiation, and it is modulated by environmental signals. Hence, development is the outcome of coupled GRN *dynamics*, spatial cues and mechano-elastic fields or constrains without the need of a central “choreographer” (Barrio et al., 2010). Here, we focused on multi-stability as an emergent property of the FT-GRN that explains the attainment of different types of meristems at the apex, and differentiation in response to endogenous and environmental signals during plant development from vegetative to reproductive states. The FT-GRN constitutes an important step toward the systems biology understanding of *A. thaliana* reproductive development, and it can be coupled with cellular dynamics of a growing domain and with explicit spatial components in the future to further understand additional morphogenetic patterns and behaviors.

### Limitations of the model and perspectives

Although the present FT-GRN model recapitulates phase transitions of the SAM, it has limitations. It does not include some signals known to participate in the reproductive phase transition, such as changes in temperature (Susila et al., 2018) trehalose-6-phosphate signaling (Wahl et al., 2013), other hormones besides GA, or redox status (Huang et al., 2021).

Also, we did not include chromatin remodeling factors, implicated in SAM’s development (You et al., 2017), which may add another level of regulatory complexity to the FT-GRN network. In a study that analyzed genome-wide histone modifications by the distribution of H3K27me3 and H3K4me3 marks along with expression changes in different developmental series including flowering transition in *A. thaliana*, it is reported that during early flower morphogenesis, changes in H3K4me3 prevail over changes in H3K27me3 and quantitatively correlate with expression changes, while H3K27me3 changes occur later (Engelhorn et al., 2017). Also, they found that genes with higher levels of H3K4me3 and lower levels of H3K27me3 are more prone to coincide with expression changes. Two MADS-box genes *SEPALLATA 1* and *2* (*SEP1/2*) have that characteristic. Furthermore, it has been proved that AP1 and SEP3 bind and help to open the chromatin before expression changes are observed at the apical meristem during reproductive transition (Engelhorn et al., 2017). Further functional analyses of these genes and their epigenetic regulation may be key to incorporate an upper-level of regulation in the FT-GRN.

Moreover, temporal patterns of the epigenetic marks are likely to vary among cell-types at the SAM. The expression patterns observed in different cell types by scRNA-seq (Zhang et al., 2021) can be related to many functions of each gene besides SAM’s phase transition. In fact, all genes detected in the aerial part of the plant are found in the root as well (Supplementary Table 6). Such complex expression patterns emphasize the need of regulatory multi-stable network models to integrate available functional data, postulate a network-level mechanistic model and validate the recovered attractors with single-cell top-down data. Nonetheless, for now we may say that many of the genes of the network expressed in both the RAM and SAM showed expression at the stem cells and/or the proliferation regions of the meristems, consistent with their role in generic meristem maintenance and cellular differentiation decisions at the root and the shoot.

Single-cell RNA sequencing is a powerful technique to be considered. The information given by this technique shows that *FCA* and *PNY* (to which there were no information at the vegetative stage) are expressed in a diverse set of cell types. Additional functional experiments are required to understand their regulation at the vegetative stage. It also helps us to postulate new hypothesis. SOC1 and AGL24 proteins heterodimerize allowing SOC1 to enter the nucleus (Lee et al., 2008). However, differential expression patterns given by scRNA-seq does not coincide completely with this idea, neither at the aerial part or the root cell types. Although further research is required, we may speculate that, similarly to FLC and SVP (Mateos et al., 2015), SOC1 and AGL24 may have joint and independent functions, at least for those vegetative stages. It is known that some of the proteins are able to migrate and probably the same happens between cell types. Thus, the expression results need to be taken with caution. Nevertheless, it would be important to compare the data from single cell transcriptomic analyses from inflorescence meristem with the data of the vegetative phase to find out if those expression patterns in each cell-type persist or if they change along development.

Additional new findings may be relevant to complement the network to understand SAM’s phase transitions. For example, it has been discovered that FLC/SVP bind and represses *TARGET OF FLC AND SVP 1* (*TFS1*), while SOC1/SPL9 upregulates it in the presence of different chromatin remodeling factors (Richter et al., 2019; Madrid et al., 2021). Additionally, it has been reported that GA signaling participates in flowering transition under lower mild temperatures, independently of *FT* regulation (Galvão et al., 2015), and also by repression of flowering repressors via GAI ASSOCIATED FACTOR 1 (GAF1; Fukazawa et al., 2021). The present model can be modified as new interactions are updated. Finally, it would be interesting to include genes implicated in stem cell maintenance and the genes that regulate them during meristem termination, for example, *WUSCHEL* (*WUS*), *CLAVATA* (*CLV*), and *AGAMOUS* (*AG*; Landau et al., 2015).

## Data availability statement

The original contributions presented in the study are publicly available. This data can be found here: https://github.com/CaroChavez/FT-GRN.

## Author contributions

EC-H performed the data curation and network modeling. SQ obtained the novel experimental results. EÁ-B contributed to the responsibility for the central theorical-conceptual approach for the construction of the network models underlying the apical meristems. BG-P contributed to the formalization of experimental data and helped integrate the logical rules. EÁ-B and BG-P obtained funding and supervised the work. EÁ-B, BG-P, and EC-H prepared the manuscript. All authors reviewed and approved the submitted version.
