# Supplementary material for: The flowering transition pathways converge into a complex gene regulatory network that underlies the phase changes of the shoot apical meristem in Arabidopsis thaliana
Source: Front Plant Sci. 2022 Aug 9;13:852047. doi: 10.3389/fpls.2022.852047 (PMC9396034; doi:10.3389/fpls.2022.852047)
Supplement: Supplementary file 2 [file Data_Sheet_1.PDF]

Supplementary Figures

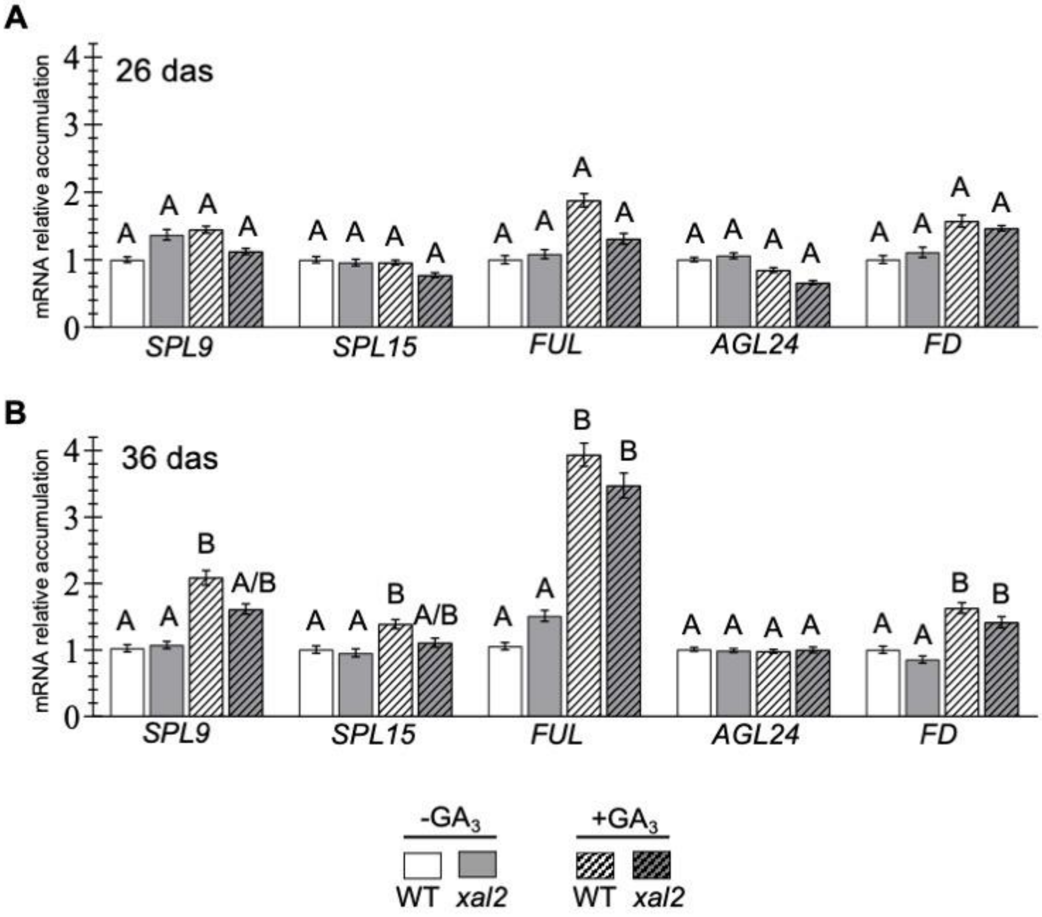

Supplementary Figure 1. Transcript relative accumulation of different genes in wild-type plants (WT) and *xal2* mutant in response to GA_3. **(A)** Shoot apices of 26 das plants and **(B)** 36 das plants of both genotypes were treated with GA_3. Differences were not significant for these genes to be included in the model. However, there was a slight reduction tendency in *SPL9*, *SPL15*, *FUL* and *FD* mRNA relative accumulation in response to the hormone in *xal2* mutant in 36 das plants. Data represent the mean value ± standard error. Statistically analysis was performed by One-Way ANOVA ($p<0.05$), followed by a Holm-Sidak's Multiple Comparison Test (3 biological replicates with 8 plants each).

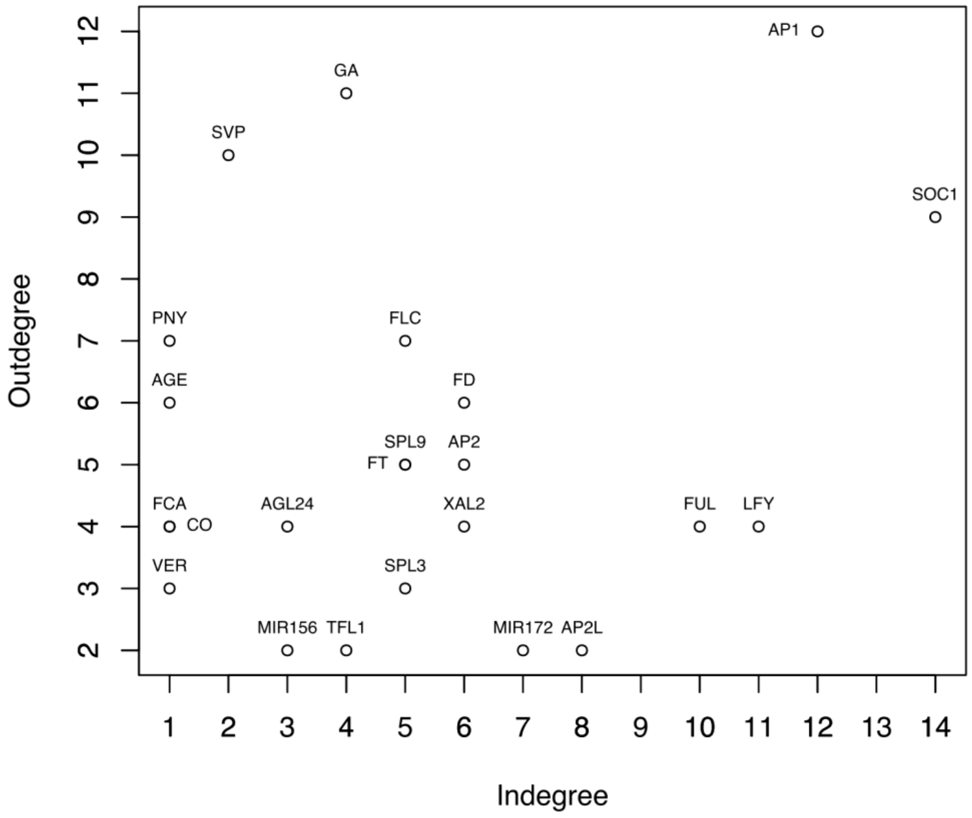

Supplementary Figure 2. FT-GRN node's indegree and outdegree plot shows that some FT-GRN nodes are more connected than others.

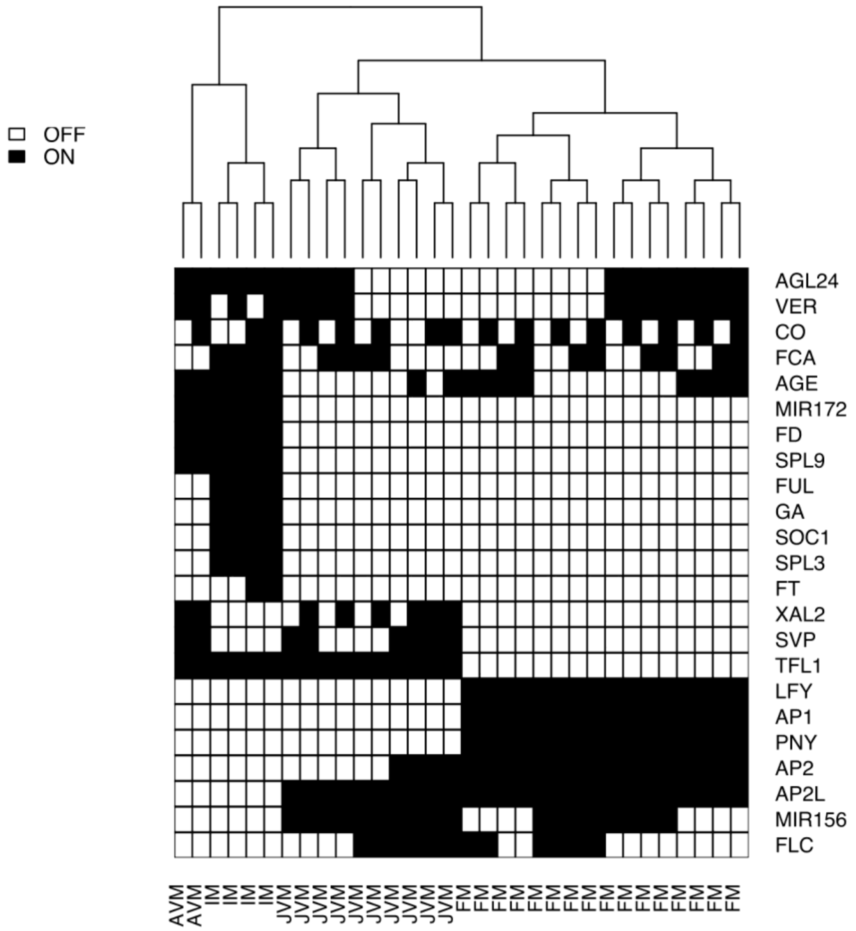

Supplementary Figure 3. The 32 attractors recovered by the model can be classified in four different phenotypes: Juvenile Vegetative Meristem (JVM), Adult Vegetative Meristem (AVM), Inflorescence Meristem (IM) and Floral Meristem (FM). Node state can be active (ON, black); or not expressed/inactive (OFF, white).

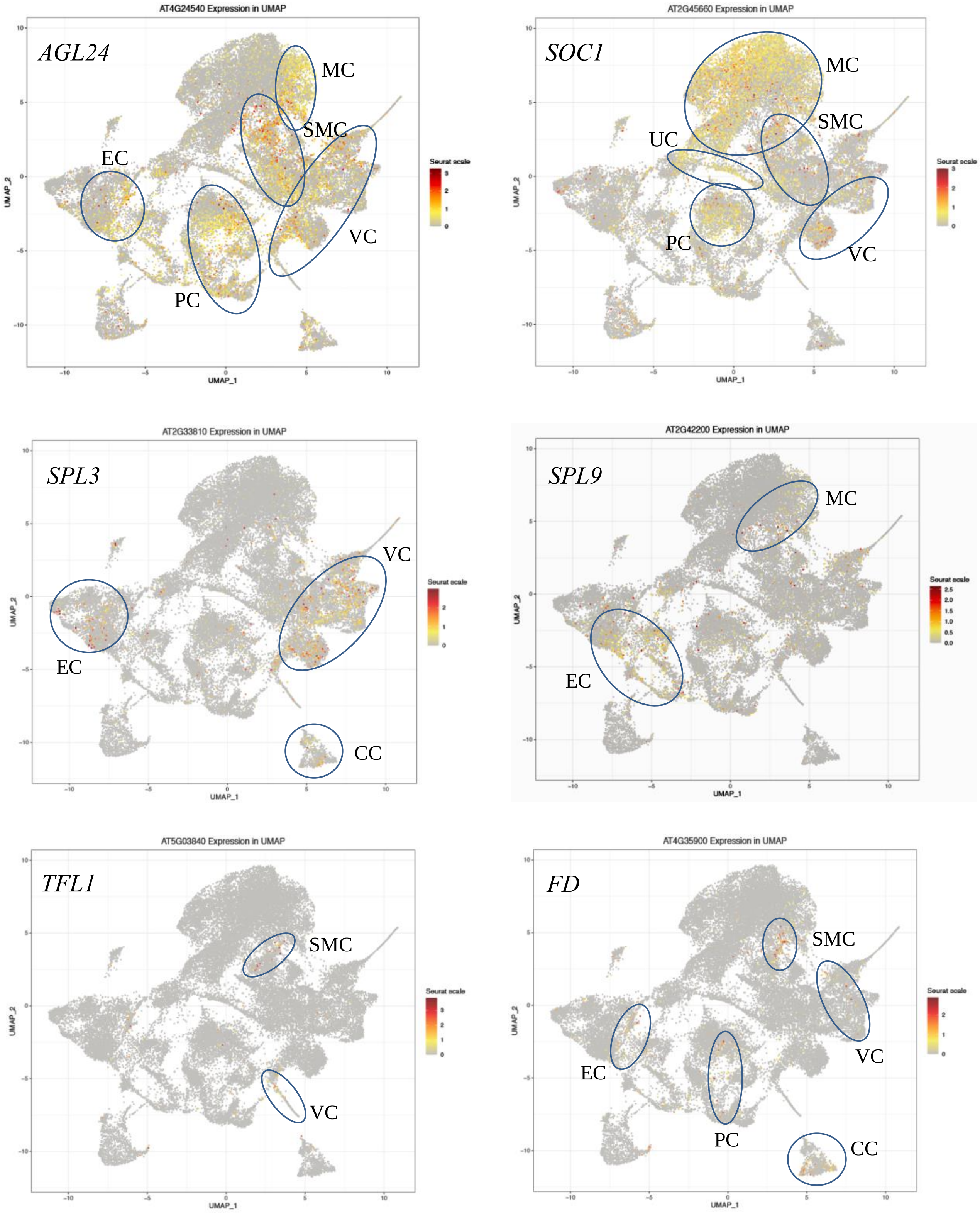

Supplementary Figure 4. Expression of six FT-GRN genes visualized by the UMAP-map obtained from http://wanglab.sippe.ac.cn/shootatlas/. Companion cell (CC), epidermal cell (EC), mesophyll cell (MC), proliferating cell (PC), shoot meristematic cell (SMC), undefined cell (UC), and vascular cell (VC) types.

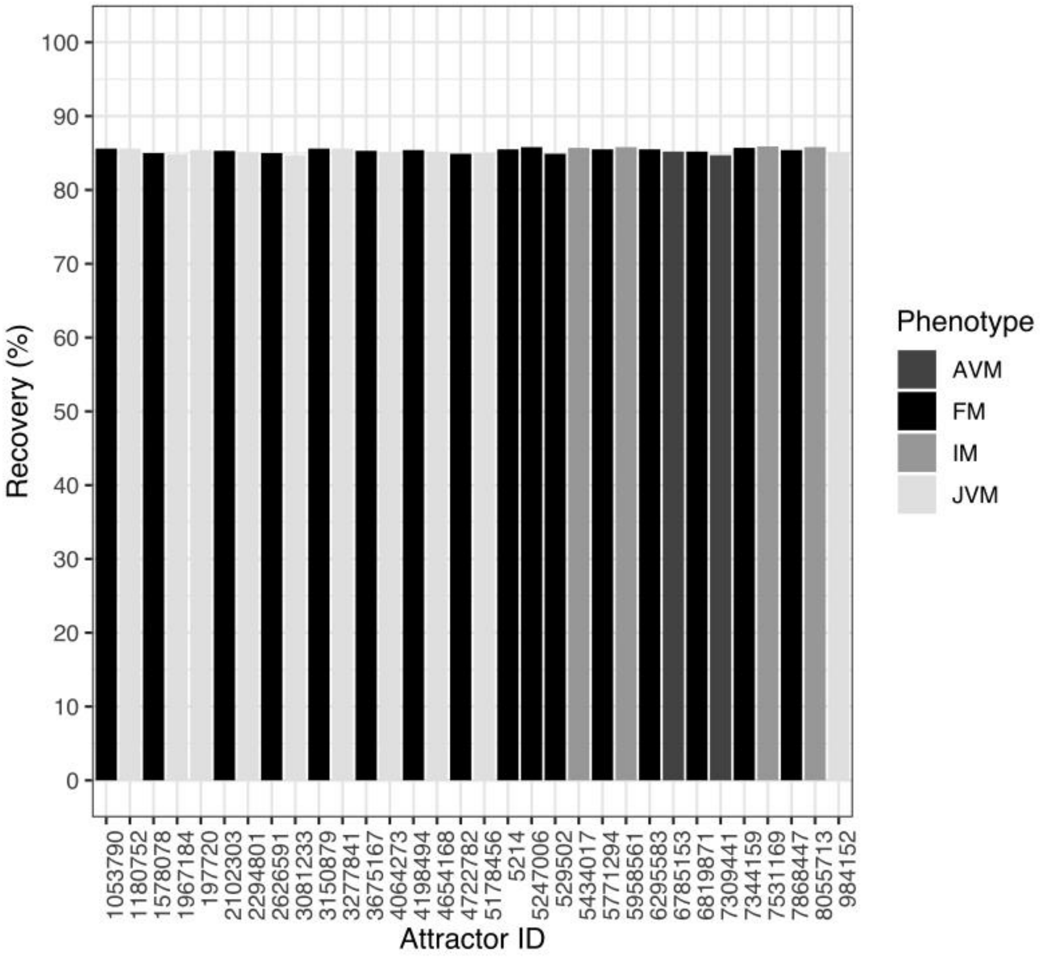

Supplementary Figure 5. Attractor's robustness observed after random perturbations were applied to the Boolean functions. 3000 perturbed models were created with one-bit random perturbation to a Boolean function. The recovery of each of the 32 original attractors (Attractor ID) in the perturbed models was calculated and colored as the corresponding phenotype.

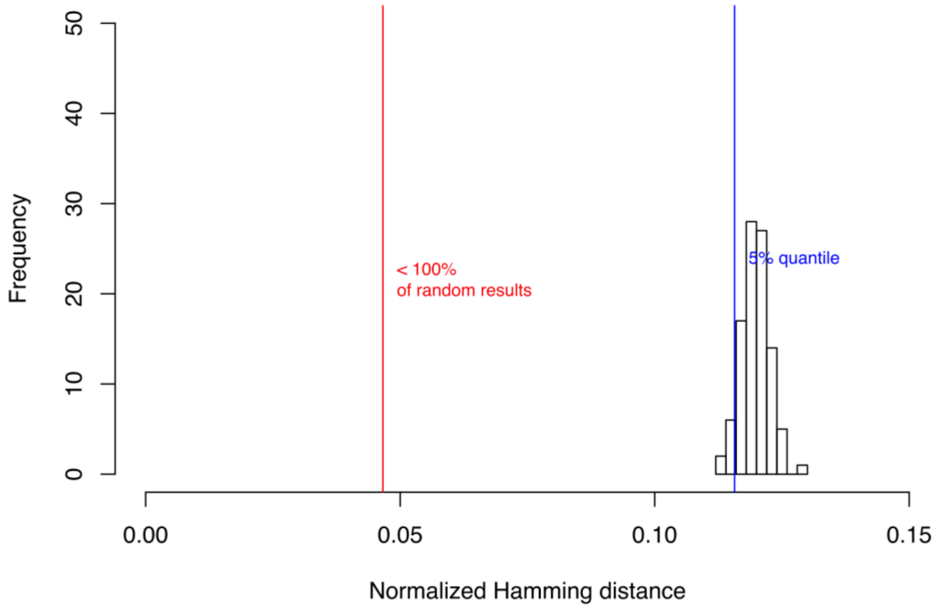

Supplementary Figure 6. The FT-GRN model is considerably more robust than random models with the same topological properties. The normalized hamming distance (used as a measure of dissimilarity between the successor states of perturbed trajectories in the state transition graph) is significantly smaller in the randomly perturbed model (red left line) than in random generated models with the same topological properties (histogram).

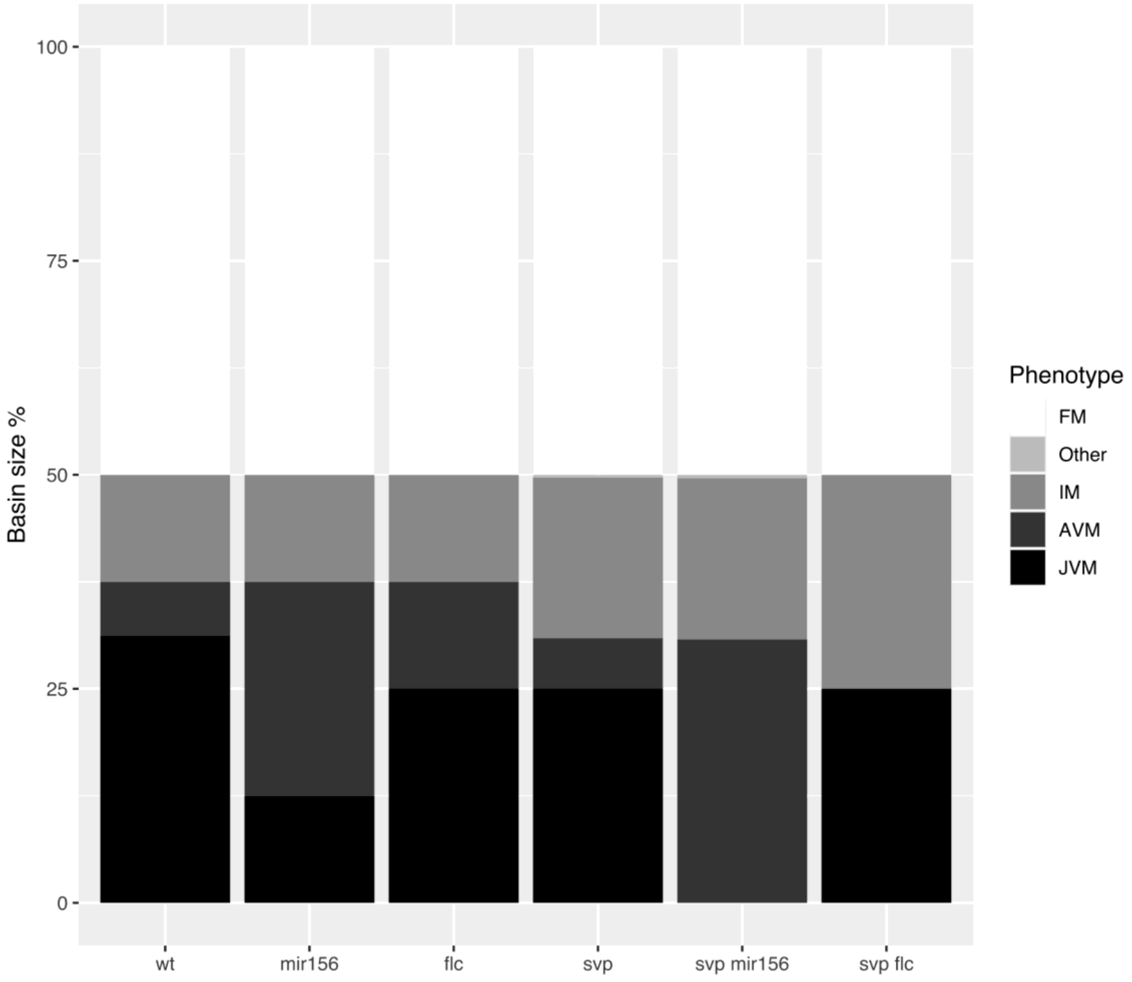

Supplementary Figure 7. Simulated double mutants suggest flowering repressors have partial redundant functions. The attractors of wild-type plants, *miR156, flc*, and *svp* single and double mutants were classified in JVM, AVM, IM, FM, and “Other” (which was a cyclic AVM/IM state in the *svp* and the *svp mir156* mutants) meristem phenotypes. The relative basin size is plotted as a percentage of all the initial state space. As expected, the introduction of *svp* mutation in the *flc* and *mir156* background increased their IM basin size, showing their redundant roles as flowering repressors.

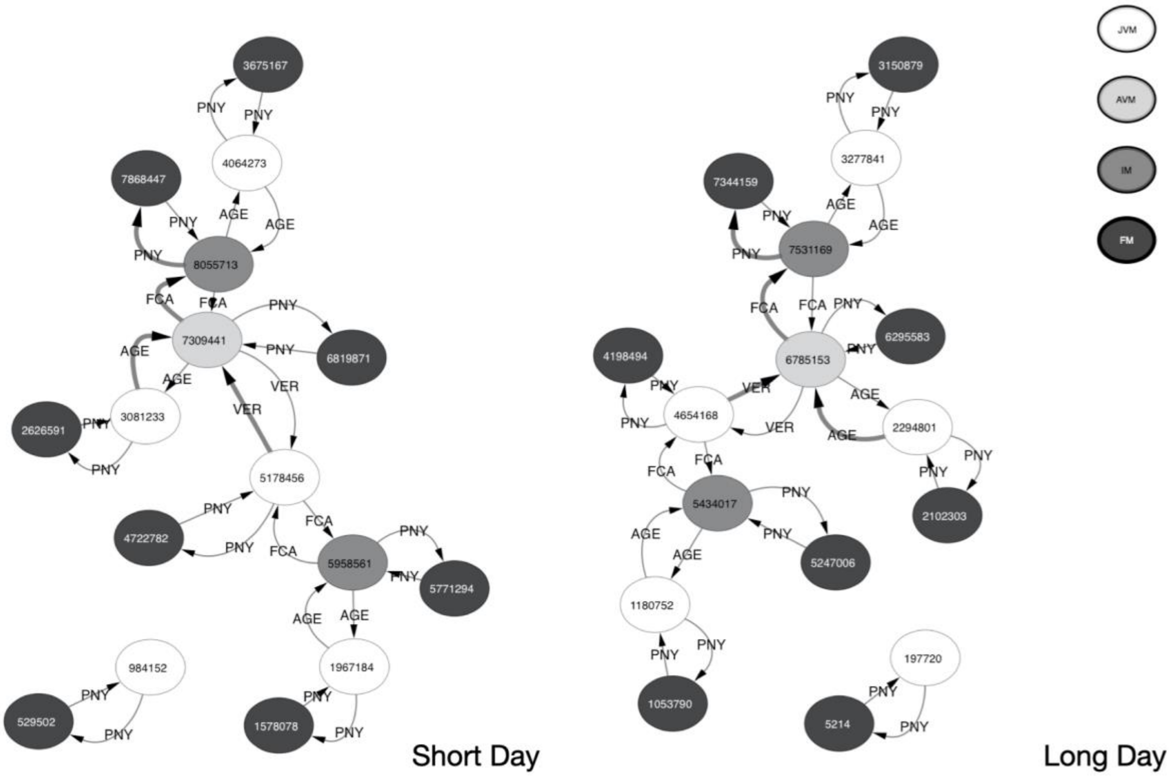

Supplementary Figure 8. Phenotype transitions can be controlled by changes in the FT-GRN input nodes. The scheme represents jumps between basins of attraction when a node of the FT-GRN model is randomly perturbed by one-bit flip in one time step starting from an attractor state. Only transitory perturbations in the input nodes (AGE, VER, FCA and PNY) can drive the system dynamics from one attractor to another with a different phenotype. Ovals represent basin of attraction of one of the 32 FT-GRN wild-type attractors, colors represent the phenotypes and numbers correspond to the attractors IDs. Arrows represent transitions from one basin of attraction to another when a FT-GRN node is perturbed, the thickest lines show trajectories observed in development.

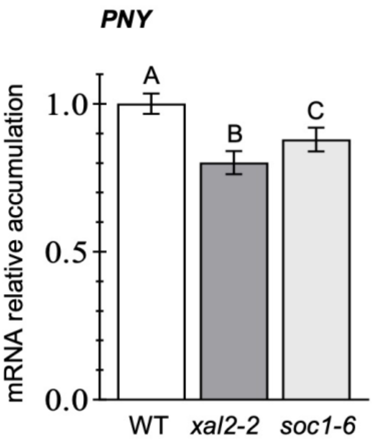

Supplementary Figure 9. *PNY* is positively regulated by XAL2 and SOC1. *PNY* mRNA relative accumulation is significantly reduced in *xal2-2* and *soc1-6,* compared to WT apices in 36 das plants grown in SD photoperiod. Data represent the mean value ± standard error. Statistically significant differences were detected by One-way ANOVA ($p<0.001$), followed by a Holm-Sidak's Multiple Comparison Test (3 biological replicates with 8 plants each).
