# Supplementary material for: The flowering transition pathways converge into a complex gene regulatory network that underlies the phase changes of the shoot apical meristem in Arabidopsis thaliana
Source: Front Plant Sci. 2022 Aug 9;13:852047. doi: 10.3389/fpls.2022.852047 (PMC9396034; doi:10.3389/fpls.2022.852047)
Supplement: Supplementary file 3 [file Data_Sheet_2.PDF]

Supplementary Table 1. Primers used in this study

XAL2-F	5’GTAGAAAGATATCAAAAGCGAA 3´	(Pérez-Ruiz et al., 2015)
XAL2-R	5’GGAGGAAACTTTTTGAAGTGT 3´	
TUB2-F	5’AGGACTCTCAAACTCACTACC3´	(Pérez-Ruiz et al., 2015)
TUB2-R	5’TCACCTTCTTCATCCGCTGTT3´	
XAL2-qF	5’GATAATTCACAGCAATCGAAGG3´	(Pérez-Ruiz et al., 2015)
XAL2-qR	5’GGTTCTCCAATTGTTGTAACTC3´	
SOC1-qF	5’AGCTGCAGAAAACGAGAAGCTCTCG3´	(Liu et al., 2008)
SOC1-qR	5’GGGCTACTCTCTTCATCACCTCTTCC3´	
LFY-qF	5’ATCGCTTGTCGTCATGGCTG3´	(Han et al., 2008)
LFY-qR	5’GCAACCGCATTGTTCCGCTC3´	
AP1-qF	5’CATGGGTGGTCTGTATCAAGAAGAT3´	(Liu et al., 2008)
AP1-qR	5’CATGCGGCGAAGCAGCCAAGGTT3´	
SPL9-qF	5’GGAATTTGACCTAGAGAAAAG3´	
SPL9-qR	5’CACCATTTTCGTAAAGCGAAG3´	
SPL15-qF	5’CAGCCACCGCCCATTTCAAC3’	(Wei et al., 2012)
SPL15-qR	5’GGAAATCTGCTGGCTCCGAGA3’	
FD-qF	5’CTCAAGAGACAACAAGATCAG3’	
FD-qR	5’CACTTCTTCATGAGACAATCTC3’	
FUL-qF	5’GTTCTTCTGCCTCAATACTG3’	
FUL-qR	5’GAGATAGTTCTACTCGTTCGT3’	
PNY-qF	5’TGCTCCTGTTTGGAGACCG3’	
PNY-qR	5’TCTGTTGGATAAGGATGCAAG 3’	
AGL24-qF	5’GAGGCTTTGGAGACAGAGTCGGTGA3’	(Liu et al., 2008)
AGL24-qR	5’AGATGGAAGCCCAAGCTTCAGGGAA3’	
PDF2-qF	5’TAACGTGGCCAAAATGATGC3´	(Czechowski et al., 2005)
PDF2-qR	5’GTTCTCCACAACCGCTTGGT3´	
RNAH-qF	5’CCATTCTACTTTTTGGCGGCT3´	(Czechowski et al., 2005)
RNAH-qR	5’TCAATGGTAACTGATCCACTCTGATG3´	
UPL7-qF	5’TTCAAATACTTGCAGCCAACCTT3´	(Czechowski et al., 2005)
UPL7-qR	5’CCCAAAGAGAGGTATCACAAGAGACT3´	

References

Czechowski, T., Stitt, M., Altmann, T., Udvardi, M. K., and Scheible, W. R. (2005). Genome-wide identification and testing of superior reference genes for transcript normalization in arabidopsis. *Plant Physiology* 139, 5–17. doi:10.1104/pp.105.063743.

Han, P., García-Ponce, B., Fonseca-Salazar, G., Alvarez-Buylla, E. R., and Yu, H. (2008). AGAMOUS-LIKE 17, a novel flowering promoter, acts in a FT-independent photoperiod pathway. *The Plant journal : for cell and molecular biology* 55, 253–265. doi:10.1111/j.1365-313X.2008.03499.x.

Liu, C., Chen, H., Er, H. L., Soo, H. M., Kumar, P. P., Han, J.-H., et al. (2008). Direct interaction of AGL24 and SOC1 integrates flowering signals in Arabidopsis. *Development* 135, 1481–1491. doi:10.1242/dev.020255.

Pérez-Ruiz, R. V, García-Ponce, B., Marsch-Martínez, N., Ugartechea-Chirino, Y., Villajuana-Bonequi, M., de Folter, S., et al. (2015). XAANTAL2 (AGL14) Is an Important Component of the Complex Gene Regulatory Network that Underlies Arabidopsis Shoot Apical Meristem Transitions. *Molecular plant* 8, 796–813. doi:10.1016/j.molp.2015.01.017.

Wei, S., Gruber, M. Y., Yu, B., Gao, M. J., Khachatourians, G. G., Hegedus, D. D., et al. (2012). Arabidopsis mutant sk156 reveals complex regulation of SPL15 in a miR156-controlled gene network. *BMC Plant Biology* 12, 169. doi:10.1186/1471-2229-12-169.
