# Supplementary material for: The flowering transition pathways converge into a complex gene regulatory network that underlies the phase changes of the shoot apical meristem in Arabidopsis thaliana
Source: Front Plant Sci. 2022 Aug 9;13:852047. doi: 10.3389/fpls.2022.852047 (PMC9396034; doi:10.3389/fpls.2022.852047)
Supplement: Supplementary file 4 [file Data_Sheet_3.PDF]

Supplementary Table 2. Node description, genes IDs and network interactions

Node	Gene(s)	Gene(s) ID	Description	Network Interactions
AGE			This node represents the adult age of the plant. The expression of MIR156 decreases as it ages. While GA levels and FD expression increase with age. FLC autonomous silencing also occurs after the plant has grown for certain time. Network input node.	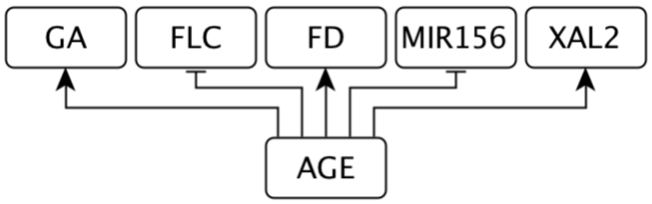
AGL24	AGL24	AT4G24540	AGAMOUS-LIKE24 (AGL24) is a MADS box TF sensitive to vernalization and gibberellin. Dimer formation with SOC1 translocates to the nucleus. Involved in flowering transition and early floral meristem development.	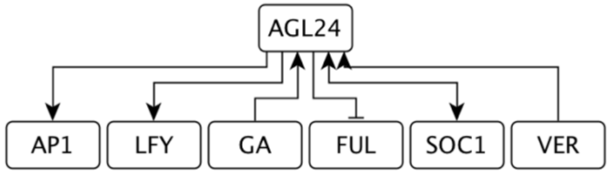
AP1	AP1<br>CAL	AT1G69120<br>AT1G26310	APETALA1 (AP1) and CAULIFLOWER (CAL) are paralogous MADS box TF that control the onset of flower development. AP1 and LFY are floral specification genes. AP1 is also necessary for the formation of the outer whorl organs of the flower.	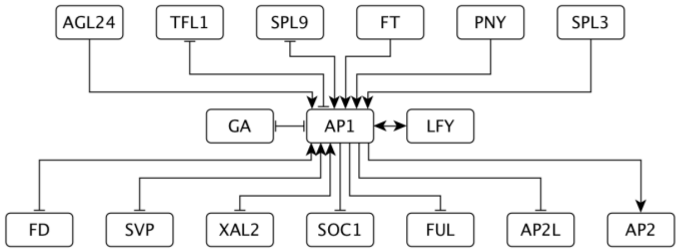

Node	Gene(s)	Gene(s) ID	Description	Network Interactions
AP2	AP2	AT4G36920	APETALA2 is a TF targeted by microRNA172 negative regulation. It can redundantly act as a repressor of flowering with AP2-like TF proteins.	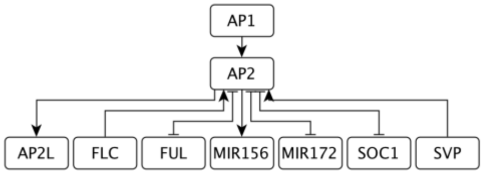
AP2L	SMZ<br>SNZ<br>TOE1<br>TOE2<br>TOE3	AT3G54990<br>AT2G39250<br>AT2G28550<br>AT5G60120<br>AT5G67180	SCHNARCHZAPFEN (SNZ), SCHLAFMUTZE (SMZ), TARGET OF EARLY ACTIVATION TAGGED (EAT) 1/2/3 (TOE1-3) are part of the family of AP2-like TF targeted by the microRNA172. They are repressors of flowering.	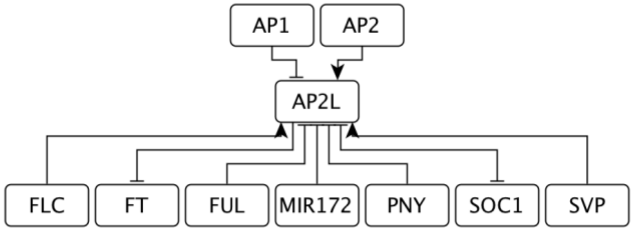
CO	CO	AT5G15840	CONSTANS (CO) is a protein similar to a zinc-finger TF, it is regulated by the length of the photoperiod. Induces SOC1, FT, and XAL2 in long days. Network input node.	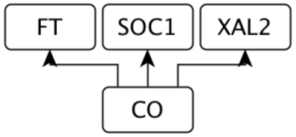
FCA	FCA	AT4G16280	FLOWERING CONTROL LOCUS A (FCA) is an RNA binding protein involved in the post-transcriptional regulation of MIR172 and SVP; and in the epigenetic silencing of FLC. FCA promotes the transition to the reproductive phase by endogenous cues independently of photoperiod length. Network input node.	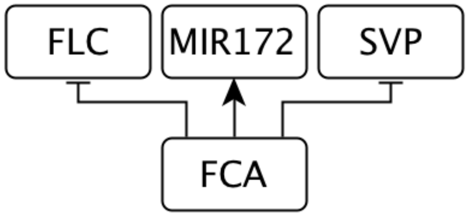

Node	Gene(s)	Gene(s) ID	Description	Network Interactions
FD	FD<br>FDP	AT4G35900<br>AT2G17770	FD and FD PARALOG (FDP) are bZIP proteins. They form a transcriptional regulatory complex with FT/TFL1 and SPL's proteins. They are necessary for FT and TFL1 activity.	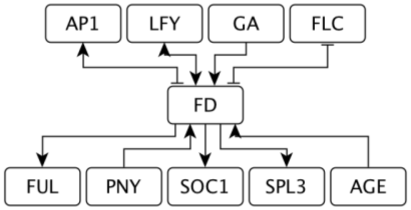
FLC	FLC	AT5G10140	FLOWERING LOCUS C (FLC) is a MADS TF repressor of flowering. FLC is repressed by sustained winter cold (vernalization) and autonomous processes.	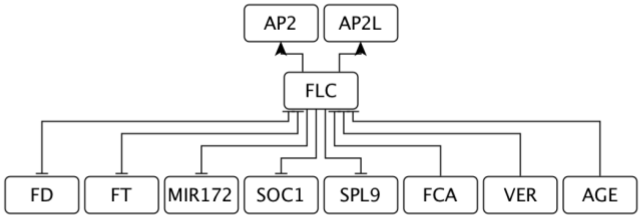
FT	FT<br>TSF	AT1G65480<br>AT4G20370	FLOWERING LOCUS T (FT) and TWIN SISTER OF FT (TSF) proteins show homology with phosphatidylethanolamine-binding proteins (PEBPs). FT is a systemic signal that travels from the leaves to the SAM in long days. It forms a complex with FD and SPL proteins. It is a positive regulator of AP1, FUL and SOC1. It counteracts activity of TFL1 and represses FLC together with FD.	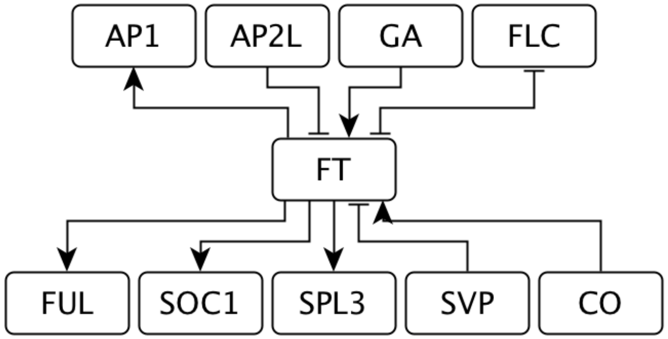

Node	Gene(s)	Gene(s) ID	Description	Network Interactions
FUL	FUL	AT5G60910	FRUITFULL (FUL) is a MADS TF involved in the prevention of secondary growth and longevity together with SOC1. Promotes reproductive development. It is involved in SAM maintenance and fruit development and patterning.	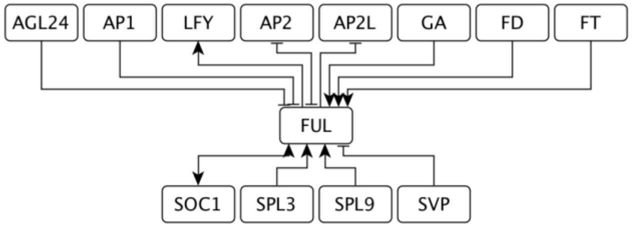
GA	GA1<br>GAI<br>RGA<br>RGL1<br>RGL2<br>RGL3<br>ELA1<br>GA20ox2<br>SPY	AT4G02780<br>AT1G14920<br>AT2G01570<br>AT1G66350<br>AT3G03450<br>AT5G17490<br>AT5G24910<br>AT5G24910<br>AT3G11540	Gibberellin (GA) related genes. REPRESSOR OF GA (RGA), RGA-LIKE1/2/3 (RGL1-3) and GIBBERELLIC ACID INSENSITIVE (GAI) are DELLA proteins degraded by GA signaling and act as transcriptional cofactors. DELLA proteins modify the transcriptional activity of SPLs, FLC and CO. EUI-LIKE P450 A1 (ELA1) codes for a GA catabolism protein induced by LFY and AP1 in the floral meristems. Gibberellin 20 oxidase 2 (GA20ox2) is a GA biosynthetic enzyme repressed in the SAM by SVP. GA REQUIRING 1 (GA1) and SPINDLY (SPY) loss of function mutants show GA reduced biosynthesis and GA overdose phenotype respectively.	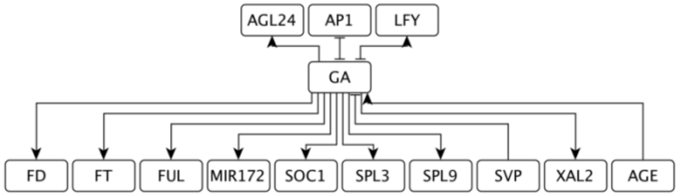

Node	Gene(s)	Gene(s) ID	Description	Network Interactions
LFY	LFY	AT5G61850	LEAFY (LFY) is a TF important for floral meristem establishment and identity in synergy with AP1. It is expressed in leaf primordia and in floral meristems. It is a direct transcriptional regulator of TFL1 and FD. It is up-regulated in the flowering transition by GA, SOC1, and FD-SPL3. It can also be regulated by FUL, SVP and AGL24. LFY and AP1 up-regulate each other in the FM. It up-regulates MADS-box genes important for floral organ formation. LFY induces a GA catabolic (ELA1) gene in the FM.	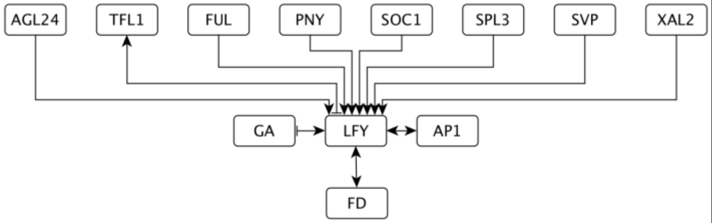
MIR156	MIR156A<br>MIR156B<br>MIR156C<br>MIR156E	AT2G25095<br>AT4G30972<br>AT4G31877<br>AT5G11977	MICRORNA 156 (MIR156) are negative regulators of the SPL TF's. miR156 and miR172 show a negative correlation in vegetative to adult development. miR156 is abundant in vegetative plants and its level is reduced as the plant grows older.	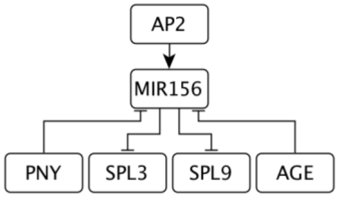
MIR172	MIR172A<br>MIR172B<br>MIR172C	AT2G28056<br>AT5G04275<br>AT3G11435	MICRORNA 172 (MIR172) are negative regulators of a some members of the AP2 TF family. miR156 and miR172 have a negative correlation in vegetative to adult development. miR172 is expressed at low levels in vegetative plants and its levels increase as the plant grows older.	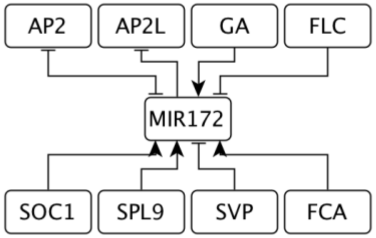

Node	Gene(s)	Gene(s) ID	Description	Network Interactions
PNY	PNF<br>PNY	AT2G27990<br>AT5G02030	POUND-FOOLISH (PNF) and PENNYWISE (PNY) are partially redundant homeodomain TFs. The double pny pnf mutant does not form flowers. PNY mutants have SAM and phyllotaxis defects. It is important for lateral organ formation and for floral organ formation. PNY and AP1 can form a transcriptional complex and they have several transcriptional targets in common. Network input node.	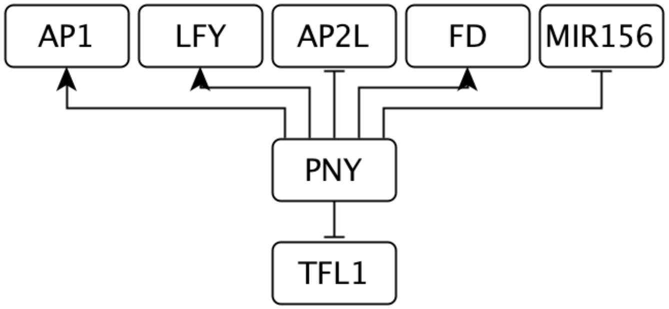
SOC1	SOC1	AT2G45660	SUPPRESSOR OF OVEREXPRESSION OF CO 1 (SOC1) is a MADS TF abundant in the IM. It is important for flowering time regulation, determinacy of the meristems, secondary vascular growth, cold responses, and senescence. It induces LFY directly. SOC1 is up-regulated in the flowering transition at the SAM by exogenous GA treatment, long day photoperiod, vernalization and aging. It is repressed by FLC, SVP, AP2-like in the vegetative phase and by AP1 and AP2 in the FM. It forms dimers with AGL24 and FUL. AGL24 dimer formation is important for SOC1 nuclear translocation.	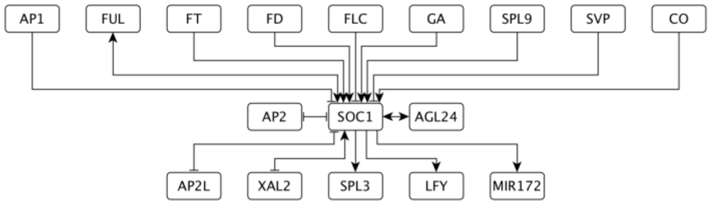

Node	Gene(s)	Gene(s) ID	Description	Network Interactions
SPL3	SPL3<br>SPL4<br>SPL5	AT2G33810<br>AT1G53160<br>AT3G15270	SQUAMOSA PROMOTER BINDING PROTEIN-LIKE (SPL) genes SPL3/4/5 are paralogous genes of the SPL TF family targeted by miR156. They form a transcriptional complex with FT and FD that up-regulates AP1, FUL and probably LFY. SPLs are inductors of adult leaf characters, they control plastochron length, flowering time and FM identity.	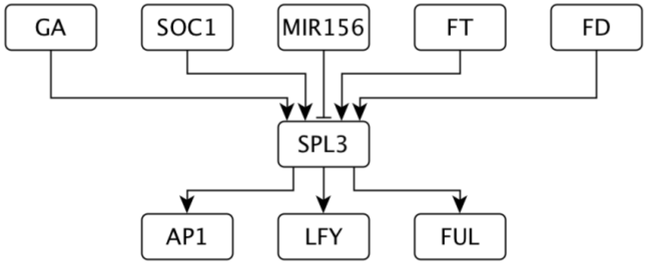
SPL9	SPL15<br>SPL9	AT3G57920<br>AT2G42200	SQUAMOSA PROMOTER BINDING PROTEIN-LIKE (SPL) SPL9/15 form a clade of paralogous genes of the SPL TF family targeted by miR156. They control flowering time, meristem identity, adult leaf characters and plastochron length. Their molecular function is regulated by DELLA proteins that are degraded by GA. SPL9/15 and SOC1 cooperatively induce FUL and MIR172.	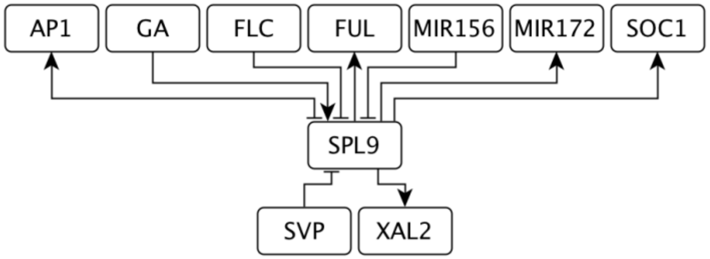

Node	Gene(s)	Gene(s) ID	Description	Network Interactions
SVP	SVP	AT2G22540	SHORT VEGETATIVE PHASE (SVP) is a MADS TF regulated by autonomous processes and temperature. SVP is a repressor of the transition from the vegetative to reproductive phase. It can form dimers with FLC, and they have overlapping as well as independent functions. There is a negative correlation between SVP and GA levels. SVP reduces gibberellin biosynthesis at the shoot apex by repression of GA20ox2. SVP can also form dimers with AP1 and it is important for floral meristem early development.	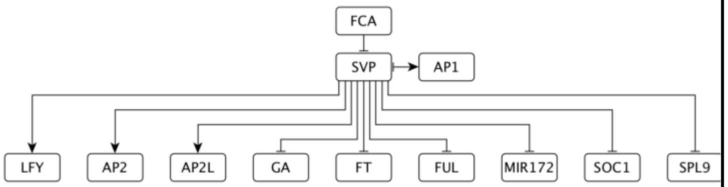
TFL1	TFL1	AT5G03840	TERMINAL FLOWER 1 (TFL1) controls meristem identity, it is expressed in vegetative meristems at low levels and up-regulated in the flowering transition in the IM and lateral meristems. It is regulated directly by AP1, XAL2 and LFY. TFL1 and FT are paralogous genes that have antagonistic functions, they both need FD to be functional.	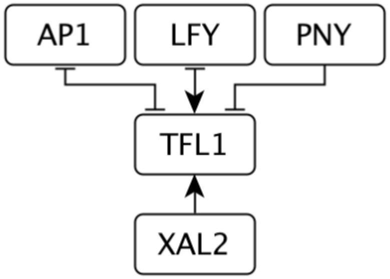

Node	Gene(s)	Gene(s) ID	Description	Network Interactions
XAL2	XAL2	AT4G11880	XAANTAL2 (XAL2) is a MADS TF, its expression increases in long days, in response to GA treatment and as the plant ages. It is a direct transcriptional activator of TFL1. Involved in the flowering transition and FM determinacy. SOC1 and AP1 are repressors of XAL2 activity. It is up-regulated by SPL9. XAL2 mediates SOC1, AP1 and LFY up-regulation in response to GA in short days.	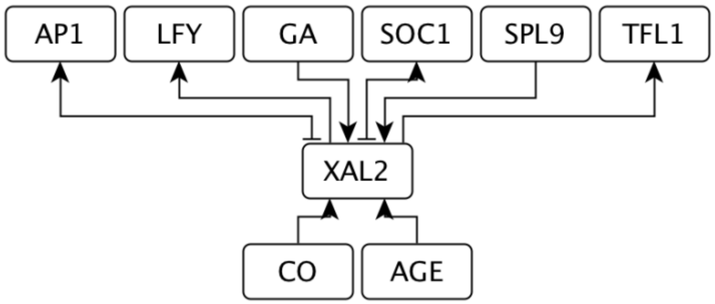
VER			Vernalization. It represents sustained winter cold. Vernalization induces flowering by repression of FLC and induction of AGL24 expression. Network input node.	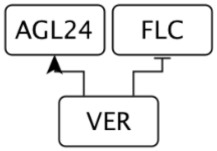
