# Supplementary material for: The flowering transition pathways converge into a complex gene regulatory network that underlies the phase changes of the shoot apical meristem in Arabidopsis thaliana
Source: Front Plant Sci. 2022 Aug 9;13:852047. doi: 10.3389/fpls.2022.852047 (PMC9396034; doi:10.3389/fpls.2022.852047)
Supplement: Supplementary file 5 [file Data_Sheet_4.PDF]

Supplementary Table 4. Logic Functions of the Boolean Model

Node	Logic Function
AGE	AGE
AGL24	(SOC1 ∧ GA) ∨ (VER)
AP1	(¬TFL1 ∨ PNY) ∧ (LFY ∨ (FT ∧ FD ∧ SPL3) ∨ (SPL9 ∧ ¬GA) ∨ (AGL24 ∧ SVP) ∨ AP1 ∨ XAL2)
LFY	¬TFL1 ∨ (PNY ∧ (GA ∨ XAL2 ∨ (SPL3 ∧ FD) ∨ AP1 ∨ (AGL24 ∧ SOC1) ∨ FUL ∨ SVP))
AP2	((SVP ∧ FLC) ∨ AP1) ∧ ¬SOC1 ∧ ¬FUL ∧ ¬MIR172
AP2L	¬MIR172 ∧ ((FLC ∧ SVP) ∨ (¬SOC1 ∧ ¬FUL ∧ ¬PNY ∧ ¬AP1) ∨ AP2)
GA	¬LFY ∧ ¬AP1 ∧ ¬SVP ∧ AGE
FLC	(¬FCA ∧ ¬VER ∧ (¬FT ∨ ¬FD))∨ (FCA ∧ ¬AGE ∧ ¬VER)
FCA	FCA
FD	¬AP1 ∧ ¬FLC ∧ (PNY ∨ LFY ∨ AGE ∨ GA)
FT	(¬FLC ∨ (FLC ∧ GA)) ∧ ¬SVP ∧ ¬AP2L ∧ CO
FUL	((FT ∧ FD ∧ SPL3) ∨ (GA ∧ SPL9) ∨ SOC1) ∧ ¬AP1 ∧ (¬AP2 ∨ (¬SVP ∧ ¬AGL24))
MIR156	¬AGE ∨ (AP2 ∧ ¬PNY)
MIR172	((¬SVP ∧ ¬FLC) ∨ ¬AP2 ∨ FCA) ∧ (SPL9 ∨ GA ∨ SOC1)
PNY	PNY
SOC1	¬SVP ∧ ¬FLC ∧ ¬AP2 ∧ ¬AP2L ∧ ¬AP1 ∧ GA ∧ (CO ∨ (FT ∧ FD) ∨ SPL9 ∨ (SOC1 ∧ AGL24) ∨ XAL2 ∨ FUL)
SPL3	¬MIR156 ∧ (SOC1 ∨ (FT ∧ FD) ∨ GA)
SPL9	(¬SVP ∨ ¬FLC) ∧ ¬MIR156 ∧ (¬AP1 ∨ GA)
SVP	¬AP1 ∧ ¬FCA
TFL1	XAL2 ∨ (LFY ∧ ¬AP1) ∨ ¬PNY
XAL2	(CO ∨ GA ∨ SPL9 ∨ AGE) ∧ ¬AP1 ∧ ¬SOC1
VER	VER
CO	CO

Boolean operators: and (∧), not (¬), or (∨).
