# Supplementary material for: The flowering transition pathways converge into a complex gene regulatory network that underlies the phase changes of the shoot apical meristem in Arabidopsis thaliana
Source: Front Plant Sci. 2022 Aug 9;13:852047. doi: 10.3389/fpls.2022.852047 (PMC9396034; doi:10.3389/fpls.2022.852047)
Supplement: Supplementary file 6 [file Data_Sheet_5.PDF]

Supplementary Table 5. Reported spatiotemporal expression data in wild-type plants

	JVM	AVM	IM	FM	References
AGL24	0	1	1	1	(Yu et al., 2002; Gregis et al., 2009)
AP1	0	0	0	1	(Parcy et al., 1998; Immink et al., 2012)
AP2	1	1	0	1	(Würschum et al., 2006; Wollmann et al., 2010)
AP2-like	1	0	0	1	(Schmid et al., 2003; Jung et al., 2007, 2014; Mathieu et al., 2009)
CO	*	*	*	NI	(Takada and Goto, 2003; An et al., 2004)
GA	1	0	1	0	(Andrés et al., 2014; Yamaguchi et al., 2014)
FLC	1	0	0	NI	(Michaels and Amasino, 1999; Michaels et al., 2005; Klepikova et al., 2015)
FD	1	1	1	1	(Abe et al., 2005)
FCA	NI	NI	NI	NI	
FT	*	*	*	0	(Takada and Goto, 2003; Abe et al., 2005; Corbesier et al., 2007)
FUL	0	1	1	0	(Mandel and Yanofsky, 1995; Gu et al., 1998; Hyun et al., 2016)
LFY	0	0	0	1	(Blazquez et al., 1997; Parcy et al., 1998; Reddy, 2008)
MIR156	1	0	1	1	(Wang et al., 2008, 2009)
MIR172	0	1	1	0	(Wollmann et al., 2010; Hyun et al., 2016)
PNY	NI	NI	1	*	(Smith and Hake, 2003; Bao et al., 2004; Smaczniak et al., 2012; Bencivenga et al., 2016)
SOC1	0	0	1	*	(Samach et al., 2000; Gregis et al., 2009; Immink et al., 2012)
SPL3	0	*	1	*	(Wang et al., 2009; Wu et al., 2009; Porri et al., 2012; Xu et al., 2016)
SPL9	0	1	1	0	(Wang et al., 2009; Wu et al., 2009; Hyun et al., 2016; Xu et al., 2016)
SVP	1	1	0	1	(Hartmann et al., 2000; Gregis et al., 2009)
TFL1	*	1	1	0	(Liu et al., 2013; Baumann et al., 2015)
XAL2	NI	1	1	1	(Schmid et al., 2005; Winter et al., 2007; Garay-Arroyo et al., 2013; Pérez-Ruiz et al., 2015)

Expressed/active (1), not expressed/inactive (0). Either present or absent (*), no information (NI).

References

Abe, M., Kobayashi, Y., Yamamoto, S., Daimon, Y., Yamaguchi, A., Ikeda, Y., et al. (2005). FD, a bZIP protein mediating signals from the floral pathway integrator FT at the shoot apex. *Science* 309, 1052–6. doi:10.1126/science.1115983.

An, H., Roussot, C., Suárez-López, P., Corbesier, L., Vincent, C., Piñeiro, M., et al. (2004). CONSTANS acts in the phloem to regulate a systemic signal that induces photoperiodic flowering of Arabidopsis. *Dev. Camb. Engl.* 131, 3615–26. doi:10.1242/dev.01231.

Andrés, F., Porri, A., Torti, S., Mateos, J., Romera-Branchat, M., García-Martínez, J. L., et al. (2014). SHORT VEGETATIVE PHASE reduces gibberellin biosynthesis at the Arabidopsis shoot apex to regulate the floral transition. *Proc. Natl. Acad. Sci. U. S. A.* 111, E2760-9. doi:10.1073/pnas.1409567111.

Bao, X., Franks, R. G., Levin, J. Z., and Liu, Z. (2004). Repression of AGAMOUS by BELLRINGER in floral and inflorescence meristems. *Plant Cell* 16, 1478–89. doi:10.1105/tpc.021147.

Baumann, K., Venail, J., Berbel, A., Domenech, M. J., Money, T., Conti, L., et al. (2015). Changing the spatial pattern of TFL1 expression reveals its key role in the shoot meristem in controlling Arabidopsis flowering architecture. *J. Exp. Bot.* 66, 4769–80. doi:10.1093/jxb/erv247.

Bencivenga, S., Serrano-Mislata, A., Bush, M., Fox, S., and Correspondence, R. S. (2016). Control of Oriented Tissue Growth through Repression of Organ Boundary Genes Promotes Stem Morphogenesis. *Dev. Cell* 39, 198–208. doi:10.1016/j.devcel.2016.08.013.

Blazquez, M. A., Soowal, L. N., Lee, I., and Weigel, D. (1997). LEAFY expression and flower initiation in Arabidopsis. *Development* 124, 3835–3844.

Corbesier, L., Vincent, C., Jang, S., Fornara, F., Fan, Q., Searle, I., et al. (2007). FT protein movement contributes to long-distance signaling in floral induction of Arabidopsis. *Science* 316, 1030–3. doi:10.1126/science.1141752.

Garay-Arroyo, A., Ortiz-Moreno, E., de la Paz Sanchez, M., Murphy, A. S., Garcia-Ponce, B., Marsch-Martinez, N., et al. (2013). The MADS transcription factor XAL2/AGL14 modulates auxin transport during Arabidopsis root development by regulating PIN expression. *EMBO J.* 32, 2884–2895. doi:10.1038/emboj.2013.216.

Gregis, V., Sessa, A., Dorca-Fornell, C., and Kater, M. M. (2009). The Arabidopsis floral meristem identity genes AP1, AGL24 and SVP directly repress class B and C floral homeotic genes. *Plant J. Cell Mol. Biol.* 60, 626–37. doi:10.1111/j.1365-313X.2009.03985.x.

Gu, Q., Ferrándiz, C., Yanofsky, M. F., and Martienssen, R. (1998). The FRUITFULL MADS-box gene mediates cell differentiation during Arabidopsis fruit development. *Dev. Camb. Engl.* 125, 1509–17.

Hartmann, U., Höhmann, S., Nettesheim, K., Wisman, E., Saedler, H., and Huijser, P. (2000). Molecular cloning of SVP: a negative regulator of the floral transition in Arabidopsis. *Plant J. Cell Mol. Biol.* 21, 351–60.

Hyun, Y., Richter, R., Vincent, C., Martinez-Gallegos, R., Porri, A., and Coupland, G. (2016). Multi-layered Regulation of SPL15 and Cooperation with SOC1 Integrate Endogenous Flowering Pathways at the Arabidopsis Shoot Meristem. *Dev. Cell* 37, 254–266. doi:10.1016/j.devcel.2016.04.001.

Immink, R. G. H., Posé, D., Ferrario, S., Ott, F., Kaufmann, K., Valentim, F. L., et al. (2012). Characterization of SOC1's central role in flowering by the identification of its upstream and downstream regulators. *Plant Physiol.* 160, 433–449. doi:10.1104/pp.112.202614.

Jung, J.-H., Lee, S., Yun, J., Lee, M., and Park, C.-M. (2014). The miR172 target TOE3 represses AGAMOUS expression during Arabidopsis floral patterning. *Plant Sci. Int. J. Exp. Plant Biol.* 215–216, 29–38. doi:10.1016/j.plantsci.2013.10.010.

Jung, J.-H., Seo, Y.-H., Seo, P. J., Reyes, J. L., Yun, J., Chua, N.-H., et al. (2007). The GIGANTEA-regulated microRNA172 mediates photoperiodic flowering independent of CONSTANS in Arabidopsis. *Plant Cell* 19, 2736–48. doi:10.1105/tpc.107.054528.

Klepikova, A. V., Logacheva, M. D., Dmitriev, S. E., and Penin, A. A. (2015). RNA-seq analysis of an apical meristem time series reveals a critical point in Arabidopsis thaliana flower initiation. *BMC Genomics* 16, 466–466. doi:10.1186/s12864-015-1688-9.

Liu, C., Teo, Z. W. N., Bi, Y., Song, S., Xi, W., Yang, X., et al. (2013). A conserved genetic pathway determines inflorescence architecture in Arabidopsis and rice. *Dev. Cell* 24, 612–22. doi:10.1016/j.devcel.2013.02.013.

Mandel, M. A., and Yanofsky, M. F. (1995). The Arabidopsis AGL8 MADS box gene is expressed in inflorescence meristems and is negatively regulated by APETALA1. *Plant Cell* 7, 1763–71. doi:10.1105/tpc.7.11.1763.

Mathieu, J., Yant, L. J., Mürdter, F., Küttner, F., and Schmid, M. (2009). Repression of Flowering by the miR172 Target SMZ. *PLoS Biol.* 7, e1000148–e1000148. doi:10.1371/journal.pbio.1000148.

Michaels, S. D., and Amasino, R. M. (1999). FLOWERING LOCUS C encodes a novel MADS domain protein that acts as a repressor of flowering. *Plant Cell* 11, 949–56.

Michaels, S. D., Himelblau, E., Kim, S. Y., Schomburg, F. M., and M, A. R. (2005). Integration of flowering signals in winter-annual Arabidopsis. *PLANT Physiol.* 137, 149–156. doi:10.1104/pp.104.052811.

Parcy, F., Nilsson, O., Busch, M. A., Lee, I., and Weigel, D. (1998). A genetic framework for floral patterning. *Nature* 395, 561–566. doi:10.1038/26903.

Pérez-Ruiz, R. V., García-Ponce, B., Marsch-Martínez, N., Ugartechea-Chirino, Y., Villajuana-Bonequi, M., de Folter, S., et al. (2015). XAANTAL2 (AGL14) Is an Important Component of the Complex Gene Regulatory Network that Underlies Arabidopsis Shoot Apical Meristem Transitions. *Mol. Plant* 8, 796–813. doi:10.1016/j.molp.2015.01.017.

Porri, A., Torti, S., Romera-Branchat, M., and Coupland, G. (2012). Spatially distinct regulatory roles for gibberellins in the promotion of flowering of Arabidopsis under long photoperiods. *Development* 139, 2198–209. doi:10.1242/dev.077164.

Reddy, G. V. (2008). Live-imaging stem-cell homeostasis in the Arabidopsis shoot apex. *Curr. Opin. Plant Biol.* 11, 88–93. doi:10.1016/j.pbi.2007.10.012.

Samach, A., Onouchi, H., Gold, S. E., Ditta, G. S., Schwarz-Sommer, Z., Yanofsky, M. F., et al. (2000). Distinct roles of CONSTANS target genes in reproductive development of Arabidopsis. *Science* 288, 1613–1616. doi:10.1126/science.288.5471.1613.

Schmid, M., Davison, T. S., Henz, S. R., Pape, U. J., Demar, M., Vingron, M., et al. (2005). A gene expression map of Arabidopsis thaliana development. *Nat. Genet.* 37, 501–506. doi:10.1038/ng1543.

Schmid, M., Uhlenhaut, N. H., Godard, F., Demar, M., Bressan, R., Weigel, D., et al. (2003). Dissection of floral induction pathways using global expression analysis. *Development* 130.

Smaczniak, C., Immink, R. G. H., Muiño, J. M., Blanvillain, R., Busscher, M., Busscher-Lange, J., et al. (2012). Characterization of MADS-domain transcription factor complexes in Arabidopsis flower development. *Proc. Natl. Acad. Sci. U. S. A.* 109, 1560–5. doi:10.1073/pnas.1112871109.

Smith, H. M. S., and Hake, S. (2003). The interaction of two homeobox genes, BREVIPEDICELLUS and PENNYWISE, regulates internode patterning in the Arabidopsis inflorescence. *Plant Cell* 15, 1717–27. doi:10.1105/TPC.012856.

Takada, S., and Goto, K. (2003). Terminal flower2, an Arabidopsis homolog of heterochromatin protein1, counteracts the activation of flowering locus T by constans in the vascular tissues of leaves to regulate flowering time. *Plant Cell* 15, 2856–65. doi:10.1105/tpc.016345.

Wang, J.-W., Czech, B., and Weigel, D. (2009). miR156-regulated SPL transcription factors define an endogenous flowering pathway in Arabidopsis thaliana. *Cell* 138, 738–49. doi:10.1016/j.cell.2009.06.014.

Wang, J.-W., Schwab, R., Czech, B., Mica, E., and Weigel, D. (2008). Dual effects of miR156-targeted SPL genes and CYP78A5/KLUH on plastochron length and organ size in Arabidopsis thaliana. *Plant Cell* 20, 1231–43. doi:10.1105/tpc.108.058180.

Winter, D., Vinegar, B., Nahal, H., Ammar, R., Wilson, G. V., and Provart, N. J. (2007). An "electronic fluorescent pictograph" Browser for exploring and analyzing large-scale biological data sets. *PLoS ONE* 2, e718–e718. doi:10.1371/journal.pone.0000718.

Wollmann, H., Mica, E., Todesco, M., Long, J. A., and Weigel, D. (2010). On reconciling the interactions between APETALA2, miR172 and AGAMOUS with the ABC model of flower development. *Dev. Camb. Engl.* 137, 3633–3642. doi:10.1242/dev.036673.

Wu, G., Park, M. Y., Conway, S. R., Wang, J.-W., Weigel, D., and Poethig, R. S. (2009). The sequential action of miR156 and miR172 regulates developmental timing in Arabidopsis. *Cell* 138, 750–9. doi:10.1016/j.cell.2009.06.031.

Würschum, T., Gross-Hardt, R., and Laux, T. (2006). APETALA2 regulates the stem cell niche in the Arabidopsis shoot meristem. *Plant Cell* 18, 295–307. doi:10.1105/tpc.105.038398.

Xu, M., Hu, T., Zhao, J., Park, M.-Y., Earley, K. W., Wu, G., et al. (2016). Developmental Functions of miR156-Regulated SQUAMOSA PROMOTER BINDING PROTEIN-LIKE (SPL) Genes in Arabidopsis thaliana. *PLoS Genet.* 12, e1006263–e1006263. doi:10.1371/journal.pgen.1006263.

Yamaguchi, N., Winter, C. M., Wu, M.-F., Kanno, Y., Yamaguchi, A., Seo, M., et al. (2014). Gibberellin Acts Positively Then Negatively to Control Onset of Flower Formation in Arabidopsis. *Science* 344, 638–641. doi:10.1126/science.1250498.

Yu, H., Xu, Y., Tan, E. L., and Kumar, P. P. (2002). AGAMOUS-LIKE 24, a dosage-dependent mediator of the flowering signals. *Proc. Natl. Acad. Sci. U. S. A.* 99, 16336–41. doi:10.1073/pnas.212624599.
