# Supplementary material for: The flowering transition pathways converge into a complex gene regulatory network that underlies the phase changes of the shoot apical meristem in Arabidopsis thaliana
Source: Front Plant Sci. 2022 Aug 9;13:852047. doi: 10.3389/fpls.2022.852047 (PMC9396034; doi:10.3389/fpls.2022.852047)
Supplement: Supplementary file 7 [file Data_Sheet_6.PDF]

Supplementary Table 6. Single-cell expression patterns of the FT-GRN genes in different cell-types from the shoot and root.

Gene	MC	SMC	EC	PC	VC	GC	CC	SE	UC
CO					*				
AGL24	*	**	*	**	*				
SOC1	**	*		*	*				*
FUL		*			*	*			*
XAL2			*	*					*
SPL3			**		**		*		
SPL9	*		*						
FD		**	*	*	*		*		
TFL1		**			*				
LFY		*							
PNY		**	**	*	**		*		
FLC		**	*	*	**		*		
FCA	*	*	*	*	*	*		*	
SVP	*	**	*	*	**	*	*	*	*
AP2	*	*	*	*	*				
TOE1	*	**	**	*	**	*	*		

Data obtained from http://wanglab.sippe.ac.cn/shootatlas/. Shoots from seven- and 14-days old and leaves from 18-days old plants (Zhang et al., 2021). Mesophyll cell (MC), shoot meristematic cell (SMC), epidermal cell (EC), proliferating cell (PC), vascular cell (VC), guard cell (GC), companion cell (CC), shoot endodermis (SE) and undefined cell (UC).

Gene	stem cell niche	root cap	epidermis	cortex	endodermis	vascular tissues	lateral root	root hair	undefined cell
CO						*			
AGL24							*	*	
SOC1	*	*	**	*	*	*		*	*
FUL	*					*			*
XAL2		*				*	*		*
SPL3		*				*			
SPL9							*		*
FD	*				*				
TFL1						*			
LFY		*			*	*	*		
PNY	*	**				*			
FLC						*			*
FCA	*	*	*	*	*	*	*	*	*
SVP	*	*				*			
AP2	*	*	*	*	*	*	*	*	*
TOE1		*			*	*	*	*	

Data from http://wanglab.sippe.ac.cn/rootatlas/. Root tips from 10-days old plants (Zhang et al., 2019).
