# Supplementary material for: The flowering transition pathways converge into a complex gene regulatory network that underlies the phase changes of the shoot apical meristem in Arabidopsis thaliana
Source: Front Plant Sci. 2022 Aug 9;13:852047. doi: 10.3389/fpls.2022.852047 (PMC9396034; doi:10.3389/fpls.2022.852047)
Supplement: Supplementary file 8 [file Data_Sheet_7.PDF]

Supplementary Table 7. Mutant and overexpressor plants phenotypes

**Mutant**	**Experimental phenotype**	**References**
agl24	Late flowering under long-days (LD) and short days (SD) conditions, and after vernalization treatment.	(Yu et al., 2002; Michaels et al., 2003)
ap1	*ap1-1* mutant flowers lack petals and have bract-like first whorl organs with floral buds formed in their axils.	(Irish and Sussex, 1990)
lfy	*lfy* mutants have partial transformation of flowers into inflorescences shoots. They have more secondary inflorescence shoots subtended by cauline leaves. In *lfy* mutant flowers eventually develop but they are abnormal: they are subtended by floral bracts, outermost organs are leaf-like, floral organs of strong *lfy* alleles are sepal-like or mosaic sepal/carpel. Often, secondary flowers arise from the axils of the outer organs. Some flowers have internode elongation. Strong alleles do not form stamens. Well-developed petals and stamens are only formed in weak *lfy* alleles, and more infrequently in intermediate alleles.	(Weigel et al., 1992)
ap2	The *ap2-1* mutant flower phenotype is temperature sensitive. At 16ºC and 25ºC the outer whorl has a conversion of sepals into leaves with stigmatic papillae at their tips. At 16ºC the second whorl organs range from petals to leaflike structures. While at 25ºC the second whorl organs show transformation of petals towards stamens. In short days, some *ap2* mutant lines produce flowers in the axils of first whorl organs. *ap2-12* mutants are early flowering in SD and LD.	(Komaki et al., 1988; Bowman et al., 1989; Yant et al., 2010)
smz snz toe1 toe2	Early flowering.	(Mathieu et al., 2009; Yant et al., 2010)
co	Late flowering under LD photoperiod.	(Rédei, 1962; Koornneef et al., 1991)

ga1-3	In SD conditions the *ga1-3* mutant fails to flower. Under continuous light, *ga1-3* plants are late flowering. The ga1-3 mutant plants do not form abaxial trichomes, a characteristic of adult leaves.	(Wilson et al., 1992; Telfer et al., 1997)
flc	*flc-3* that lacks *FRI* activity are early flowering under SD conditions. In winter annuals with active *FRI*, *flc-3* is also early flowering under LD conditions. The progression but not the onset of vegetative phase change is delayed by FLC. *FRI;FLC and FRI;flc-3* plants have flowering-dependent major differences in rosette leaf morphology associated with the vegetative phase change such as: leaf size, hydathode number and the distribution of abaxial trichomes. Leaf shape and the total number of abaxial trichomes are affected by *FLC* independently of its effect on flowering.	(Michaels and Amasino, 2001; Willmann and Poethig, 2011)
fca	This mutant is late flowering under both photoperiods, but it is responsive to vernalization. *fca* plants form more juvenile and adult rosette leaves.	(Koornneef et al., 1991; Telfer et al., 1997)
fd	Late flowering under LD.	(Koornneef et al., 1991; Abe et al., 2005)
ft	Late flowering under LD.	(Koornneef et al., 1991)
ful	Slightly late flowering under constant light, SD, and LD. Defects in valve elongation resulting in a short silique with small, crowded seeds. Leaves affected in shape, cell organization and vascular differentiation of the midvein.	(Gu et al., 1998; Ferrándiz et al., 2000; Melzer et al., 2008)
mir156	*35S::MIM156* plants, with reduced functionality of miR156, had very long plastochrons, they flower at the same time as control plants but with fewer number of leaves both in LD and SD. *35S::MIM156* plants produce rosette leaves with only adult morphology.	(Franco-Zorrilla et al., 2007; Wang et al., 2009; Xu et al., 2016)

mir172	*35S::MIM172* had reduced fruit size and epidermal valve defects. They are late flowering in both LD and SD.	(José Ripoll et al., 2015; Díaz-Manzano et al., 2018)
pny pnf	*pny pnf* double mutant has an altered phyllotaxis and do not form flowers even thought the SAM responded to flowering signals: they showed increased rate of leaf production, development of axially meristems, changes in leaf morphology. *pny/pny* and *PNF/pnf (pny-hemi)* can produce flowers and the inflorescence terminate in a central flower that lacked petals and produced mosaic sepal-carpel structures. The *pny* mutant has defects in internode patterning and partial loss of apical dominance but have normal floral organs. Missense mutants *blr-4* and *blr-5* form terminal carpeloid flowers.	(Smith and Hake, 2003; Bao et al., 2004; Smith et al., 2004; Yu et al., 2009; Lal et al., 2011)
soc1	Late flowering under LD and SD, and reduced sensitivity to GA effect on flowering promotion.	(Onouchi et al., 2000; Moon et al., 2003)
spl3 spl4 spl5	*spl3 spl4 spl5* triple mutant does not display a significant phenotype in flowering time both in LD and SD conditions. However, knockdown *SPL3/4/5* genes delays flowering in LD when grown in soil.	(Jung et al., 2016; Xu et al., 2016)
spl9 spl15	The double mutant *spl9 spl15* is late flowering under LD measured by both days and leaf number, while in SD is late flowering only when measured by leaf number.	(Schwarz et al., 2008; Xu et al., 2016)
svp	Reduced rosette and cauline leaf number. Early flowering in LD and SD but still sensitive to photoperiod and vernalization.	(Hartmann et al., 2000)
tfl1	Early flowering in SD and LD. A terminal floral meristem is developed instead of the indeterminate inflorescence meristem. The terminal flower is often abnormal and usually	(Shannon and Meeks-Wagner, 1991)

	lacks complete whorls of sepals and petals. Often, sepals of the terminal flower have carpelloid features.	
xal2	Delayed flowering both measured as bolting time and total leaf number in SD and slight delay in bolting time in LD without difference in leaf number. *xal2* mutants produce less cauline leaves in SD.	(Pérez-Ruiz et al., 2015)
35S::AGL24	Plants overexpressing *AGL24* are early flowering in LD and SD, but remained photoperiod responsive. *AGL24* overexpression caused floral abnormalities such as: abnormal floral organ number, greenish petals, elongation of the carpel and reduced fertility. The *35S:AGL24* formed flowers with secondary flowers without petals in the axils of the leaf-like sepals, inflorescences developed from swollen ovaries.	(Yu et al., 2002, 2004; Michaels et al., 2003; Liu et al., 2007)
35S::AP1	Early flowering under SD and LD. The indeterminate shoot apex produces a terminal flower. Lateral shoots are also converted to solitary flowers.	(Mandel et al., 1995)
35S::LFY	In the *LFY* overexpressor, secondary shoots developed as solitary flowers. *35S::LFY* plants in LD flower earlier that wild-type plants measured by their bolting time. In SD, overexpressor plants are early flowering measured both by leaf number and days after sowing.	(Weigel and Nilsson, 1995)
35S::AP2	*AP2* overexpressor plants had a delayed floral transition. *35S::AP2* plants showed normal flowers but *35S::AP2m1*, a miR172 resistant form, showed loss of floral determinacy. They had flowers with enlarged floral meristem surrounded by staminoid organs or petals. Also, *35S:AP2m3-GR* induced plants are late flowering.	(Chen, 2004; Yant et al., 2010)

35S::SMZ<br>*35S::TOE3*	*smz-D* is an activation tagging screen mutant that is late flowering in LD and slightly late flowering in SD. *35S::rSMZ*, a miR172 resistant mutant, remained vegetative trough their life. *35S::rSMZ* leaves had a crinkled phenotype and plants remained small. *toe1-1D* and *35S::TOE1* are late flowering in LD and SD. Overexpression of TOE2, SMZ or SNZ cause a late flowering phenotype. Overexpression of a miRNA resistant form of *TOE3* cause late flowering, indeterminacy of the flower, enlarged floral meristem, flowers with additional whorls of stamens and carpels, numerous flowers also showed reiterative flowers with normal first and second whorl organs and bulged carpels or increased number of stamens. While *35S::TOE3* flowers and flowering time is the same as the wild-type.	(Aukerman and Sakai, 2003; Schwab et al., 2005; Jung et al., 2007, 2014; Mathieu et al., 2009)
35S::CO	*35S::CO-GR* induced lines and *35S::CO* plants flower early and are photoperiod insensitive, the induction of CO early in development reduces the number of cauline leaves and flowers produced in the main shoot, transgenic plants have reduced stem length and smaller rosette leaves. Frequently, the main shoot is terminated with the formation of a carpeloid structure. The flowers of *35S::CO* plants often have three or four carpels instead of two.	(Simon et al., 1996; Onouchi et al., 2000)
35S::FLC	Late flowering or never flowering.	(Michaels and Amasino, 1999)
35S::FCA	*35S::FCA* in L*er* background plants are slightly early flowering in LD at 16º C, and in SD.	(Macknight et al., 1997)
35S::FD	Overexpression of *FD* produced dwarf plants with up-curled leaves and slightly early flowering.	(Abe et al., 2005)

35S::FT	Early flowering. Production of two-four rosette leaves independently of the photoperiod. The inflorescence meristem is determined in a terminal flower.	(Kardailsky et al., 1999; Kobayashi et al., 1999; Hanzawa et al., 2005)
35S::FUL	*35S::FUL* plants flower early in LD and SD, they produce terminal flowers, show an increase in seed weight, and have fruits with conversion of the valve margin cells and outer replum into valve cells, the dehiscence zone of *35S:FUL* fruits fails to form.	(Ferrándiz et al., 2000; Balanzà et al., 2014)
35S::MIR156	These plants are late flowering in LD and SD measured by days after sowing and particularly by an increased leaf number. Plants had a shorter plastochron, leaves with juvenile appearance, decrease in apical dominance and inflorescence height, first flowers often arise from side shoots, flowers with squashed appearance.	(Schwab et al., 2005; Wu and Poethig, 2006; Schwarz et al., 2008)
35S::MIR172	Plants overexpressing *MIR172* had an accelerated floral transition, some lines showed a floral phenotype with first-whorl organs transformed into carpels similar to *ap2* loss of function mutants. Transgenic plants had some cauline leaves with stigmatic papillae, and sometimes plants had upward curled leaves.	(Chen, 2004)
35S::SOC1	Overexpression of SOC1 caused early flowering in LD and SD conditions. Strong lines showed photoperiod-independent early flowering. Also, they showed more cauline leaves and leaf-like sepals specially under SD. Some early flowering transformants were sterile due to elongated pistils that prevented self-pollination, and they also had smaller and sepaloid light green petals.	(Borner et al., 2000; Samach et al., 2000; Liu et al., 2007)
spy1	Mutants in the *SPINDLY* locus resemble wild-type plants that have been repeatedly sprayed with GA_3 (GA overdose phenotype). In comparison to wild-type, *spy1* mutants	(Jacobsen and Olszewski, 1993; Telfer et al., 1997)

	exhibit longer hypocotyls, lighter green color leaves, increased stem elongation, early flowering, parthenocarpy, and partial male sterility. Both, *spy1* plants and GA_3-treated wild-type plants flowered earlier than control wild-type plants when measured either by the number of rosette leaves or bolting time. Also in both cases, trichome production was enhanced in the abaxial side of leaves (a mature vegetative trait).	
35S::SPL3	The *35S::SPL3se* line (resistant to miR156 degradation) is early flowering in LD and SD, but they are still photoperiod sensitive. Basal flowers are subtended by bracts, specially, in SD conditions. These plants flowered early, had a reduced number of juvenile, adult and cauline leaves both in SD and LD, and they had fewer secondary inflorescences. Notably, the first two leaves had features of adult leaves. Similarly, overexpression of miR156-resistant *SPL4* and *SPL5* caused early floral induction and accelerated abaxial trichome production.	(Cardon et al., 1997; Wu and Poethig, 2006; Yamaguchi et al., 2009)
SPL9::rSPL9<br>*SPL15::V9A:rSPL15*	High levels of a miR156 resistant form of *SPL9* under the *35S* promoter caused embryonic lethality. However, *SPL9::rSPL9* plants had a reduced meristem, increased plastochron length, and they had lost their juvenile phase as shown by the production of adult-specific leaf traits. Induction of *35S::GR:rSPL9* lines in the presence of dexamethasone had a similar phenotype to that of *SPL9::rSPL9* plants. Also, expression of a miR156-resistant version of *SPL15* from its own promoter showed acceleration of flowering in SD and LD.	(Wang et al., 2008; Wu et al., 2009; Hyun et al., 2016)
35S::SVP	Plants overexpressing *SVP* were late flowering in LD at 23 oC and 16 oC. *35S::SVP* flowers had vegetative characters: branched trichomes on sepals, petals and carpels and green	(Masiero, 2004; Lee et al., 2007; Liu et al., 2007)

	petals. *35S::SVP* flowers continuously developed leaves with elongated internodes in a whorled or a spiral pattern, stamens occasionally developed from the axils of leaves. The elongated *35S::SVP* flowers eventually terminated with a mixture of leaves, carpelloid leaves and stamens.	
35S::TFL1	Plants overexpressing TFL1 had an extended vegetative and inflorescence phase. Plants developed a highly branched architecture. Some *35S::TFL1* individuals in SD conditions remained in the inflorescence phase and never produced flowers. Other phenotypes observed in *35S::TFL1* plants were formation of aerial rosettes, formation of clusters of flowers surrounded by leaf like organs in a whorled pattern, flowers with organ abnormalities and additional flower buds in the axils of those organs.	(Ratcliffe et al., 1998)
35S::XAL2	*35S::XAL2* plants are early bolting, and they have fewer rosette and cauline leaves than wild type plants both in SD and LD conditions. Inflorescences grew from basipetal carpels of *XAL2* overexpressor plants grown in SD. Cauline leaves of *XAL2* overexpressor plants are large similar to rosette leaves, and flowers have large indehiscent sepals that have leaf cell morphology including branched trichomes.	(Pérez-Ruiz et al., 2015)

References

Abe, M., Kobayashi, Y., Yamamoto, S., Daimon, Y., Yamaguchi, A., Ikeda, Y., et al. (2005). FD, a bZIP protein mediating signals from the floral pathway integrator FT at the shoot apex. *Science* 309, 1052–6. doi:10.1126/science.1115983.

Aukerman, M. J., and Sakai, H. (2003). Regulation of flowering time and floral organ identity by a MicroRNA and its APETALA2-like target genes. *Plant Cell* 15, 2730–41. doi:10.1105/tpc.016238.

Balanzà, V., Martínez-Fernández, I., and Ferrándiz, C. (2014). Sequential action of FRUITFULL as a modulator of the activity of the floral regulators SVP and SOC1. *J. Exp. Bot.* 65, 1193–203. doi:10.1093/jxb/ert482.

Bao, X., Franks, R. G., Levin, J. Z., and Liu, Z. (2004). Repression of AGAMOUS by BELLRINGER in floral and inflorescence meristems. *Plant Cell* 16, 1478–89. doi:10.1105/tpc.021147.

Borner, R., Kampmann, G., Chandler, J., Gleißner, R., Wisman, E., Apel, K., et al. (2000). A MADS domain gene involved in the transition to flowering in Arabidopsis. *Plant J.* 24, 591–599. doi:10.1046/j.1365-313X.2000.00906.x.

Bowman, J. L., Smyth, D. R., and Meyerowitz, E. M. (1989). Genes directing flower development in Arabidopsis. *Plant Cell* 1, 37–52. doi:10.1105/TPC.1.1.37.

Cardon, G. H., Höhmann, S., Nettesheim, K., Saedler, H., and Huijser, P. (1997). Functional analysis of the Arabidopsis thaliana SBP-box gene SPL3: A novel gene involved in the floral transition. *Plant J.* 12, 367–377. doi:10.1046/j.1365-313X.1997.12020367.x.

Chen, X. (2004). A microRNA as a translational repressor of APETALA2 in Arabidopsis flower development. *Science* 303, 2022–5. doi:10.1126/science.1088060.

Díaz-Manzano, F. E., Cabrera, J., Ripoll, J. J., del Olmo, I., Andrés, M. F., Silva, A. C., et al. (2018). A role for the gene regulatory module microRNA172/TARGET OF EARLY ACTIVATION TAGGED 1/FLOWERING LOCUS T (miRNA172/TOE1/FT) in the feeding sites induced by Meloidogyne javanica in Arabidopsis thaliana. *New Phytol.* 217, 813–827. doi:10.1111/nph.14839.

Ferrándiz, C., Liljegren, S. J., and Yanofsky, M. F. (2000). Negative regulation of the SHATTERPROOF genes by FRUITFULL during Arabidopsis fruit development. *Science* 289, 436–438. doi:10.1126/science.289.5478.436.

Franco-Zorrilla, J. M., Valli, A., Todesco, M., Mateos, I., Puga, M. I., Rubio-Somoza, I., et al. (2007). Target mimicry provides a new mechanism for regulation of microRNA activity. *Nat. Genet.* 39, 1033–1037. doi:10.1038/ng2079.

Gu, Q., Ferrándiz, C., Yanofsky, M. F., and Martienssen, R. (1998). The FRUITFULL MADS-box gene mediates cell differentiation during Arabidopsis fruit development. *Dev. Camb. Engl.* 125, 1509–17.

Hanzawa, Y., Money, T., and Bradley, D. (2005). A single amino acid converts a repressor to an activator of flowering. *Proc. Natl. Acad. Sci. U. S. A.* 102, 7748–53. doi:10.1073/pnas.0500932102.

Hartmann, U., Höhmann, S., Nettesheim, K., Wisman, E., Saedler, H., and Huijser, P. (2000). Molecular cloning of SVP: a negative regulator of the floral transition in Arabidopsis. *Plant J. Cell Mol. Biol.* 21, 351–60.

Hyun, Y., Richter, R., Vincent, C., Martinez-Gallegos, R., Porri, A., and Coupland, G. (2016). Multi-layered Regulation of SPL15 and Cooperation with SOC1 Integrate Endogenous Flowering Pathways at the Arabidopsis Shoot Meristem. *Dev. Cell* 37, 254–266. doi:10.1016/j.devcel.2016.04.001.

Irish, V. F., and Sussex, I. M. (1990). Function of the apetala-1 gene during Arabidopsis floral development. *Plant Cell* 2, 741–53. doi:10.1105/tpc.2.8.741.

Jacobsen, S. E., and Olszewski, N. E. (1993). Mutations at the SPINDLY locus of Arabidopsis alter gibberellin signal transduction. *Plant Cell* 5, 887–896. doi:10.1105/tpc.5.8.887.

José Ripoll, J., Bailey, L. J., Mai, Q.-A., Wu, S. L., Hon, C. T., Chapman, E. J., et al. (2015). microRNA regulation of fruit growth. *Nat. Plants* 1, 15036–15036. doi:10.1038/nplants.2015.36.

Jung, J. H., Lee, H. J., Ryu, J. Y., and Park, C. M. (2016). SPL3/4/5 Integrate Developmental Aging and Photoperiodic Signals into the FT-FD Module in Arabidopsis Flowering. *Mol. Plant* 9, 1647–1659. doi:10.1016/j.molp.2016.10.014.

Jung, J.-H., Lee, S., Yun, J., Lee, M., and Park, C.-M. (2014). The miR172 target TOE3 represses AGAMOUS expression during Arabidopsis floral patterning. *Plant Sci. Int. J. Exp. Plant Biol.* 215–216, 29–38. doi:10.1016/j.plantsci.2013.10.010.

Jung, J.-H., Seo, Y.-H., Seo, P. J., Reyes, J. L., Yun, J., Chua, N.-H., et al. (2007). The GIGANTEA-regulated microRNA172 mediates photoperiodic flowering independent of CONSTANS in Arabidopsis. *Plant Cell* 19, 2736–48. doi:10.1105/tpc.107.054528.

Kardailsky, I., Shukla, V. K., Ahn, J. H., Dagenais, N., Christensen, S. K., Nguyen, J. T., et al. (1999). Activation tagging of the floral inducer FT. *Science* 286, 1962–5. doi:10.1126/SCIENCE.286.5446.1962.

Kobayashi, Y., Kaya, H., Goto, K., Iwabuchi, M., and Araki, T. (1999). A Pair of Related Genes with Antagonistic Roles in Mediating Flowering Signals. *Science* 286, 1960–1962. doi:10.1126/SCIENCE.286.5446.1960.

Komaki, M. K., Okada, K., N1shino, E., and Shimura, Y. (1988). Isolation and characterization of novel mutants of Arabidopsis thaliana defective in flower development. *Development* 104, 195–203.

Koornneef, M., Hanhart, C. J., and van der Veen, J. H. (1991). A genetic and physiological analysis of late flowering mutants in Arabidopsis thaliana. *Mol. Gen. Genet. MGG* 229, 57–66. doi:10.1007/BF00264213.

Lal, S., Pacis, L. B., and Smith, H. M. S. (2011). Regulation of the SQUAMOSA PROMOTER-BINDING PROTEIN-LIKE genes/microRNA156 module by the homeodomain proteins PENNYWISE and POUND-FOOLISH in Arabidopsis. *Mol. Plant* 4, 1123–32. doi:10.1093/mp/ssr041.

Lee, J. H., Yoo, S. J., Park, S. H., Hwang, I., Lee, J. S., and Ahn, J. H. (2007). Role of SVP in the control of flowering time by ambient temperature in Arabidopsis. *Genes Dev.* 21, 397–402. doi:10.1101/gad.1518407.

Liu, C., Zhou, J., Bracha-Drori, K., Yalovsky, S., Ito, T., and Yu, H. (2007). Specification of Arabidopsis floral meristem identity by repression of flowering time genes. *Development* 134, 1901–1910. doi:10.1242/dev.003103.

Macknight, R., Bancroft, I., Page, T., Lister, C., Schmidt, R., Love, K., et al. (1997). FCA, a gene controlling flowering time in Arabidopsis, encodes a protein containing RNA-binding domains. *Cell* 89, 737–45. doi:10.1016/S0092-8674(00)80256-1.

Mandel, M. A., Yanofsky, M., and F. (1995). A gene triggering flower formation in Arabidopsis. *Nature* 377, 522–524. doi:10.1038/377522a0.

Masiero, S. (2004). INCOMPOSITA: a MADS-box gene controlling prophyll development and floral meristem identity in Antirrhinum. *Development* 131, 5981–5990. doi:10.1242/dev.01517.

Mathieu, J., Yant, L. J., Mürdter, F., Küttner, F., and Schmid, M. (2009). Repression of Flowering by the miR172 Target SMZ. *PLoS Biol.* 7, e1000148–e1000148. doi:10.1371/journal.pbio.1000148.

Melzer, S., Lens, F., Gennen, J., Vanneste, S., Rohde, A., and Beeckman, T. (2008). Flowering-time genes modulate meristem determinacy and growth form in Arabidopsis thaliana. *Nat. Genet.* 40, 1489–1492. doi:10.1038/ng.253.

Michaels, S. D., and Amasino, R. M. (1999). FLOWERING LOCUS C encodes a novel MADS domain protein that acts as a repressor of flowering. *Plant Cell* 11, 949–56.

Michaels, S. D., and Amasino, R. M. (2001). Loss of FLOWERING LOCUS C activity eliminates the late-flowering phenotype of FRIGIDA and autonomous pathway mutations but not responsiveness to vernalization. *Plant Cell* 13, 935–41. doi:10.1105/tpc.13.4.935.

Michaels, S. D., Ditta, G., Gustafson-Brown, C., Pelaz, S., Yanofsky, M., and Amasino, R. M. (2003). AGL24 acts as a promoter of flowering in Arabidopsis and is positively regulated by vernalization. *Plant J.* 33, 867–874. doi:10.1046/j.1365-313X.2003.01671.x.

Moon, J., Suh, S.-S., Lee, H., Choi, K.-R., Hong, C. B., Paek, N.-C., et al. (2003). The SOC1 MADS-box gene integrates vernalization and gibberellin signals for flowering in Arabidopsis. *Plant J.* 35, 613–623. doi:10.1046/j.1365-313X.2003.01833.x.

Onouchi, H., Igeño, M. I., Périlleux, C., Graves, K., and Coupland, G. (2000). Mutagenesis of plants overexpressing CONSTANS demonstrates novel interactions among Arabidopsis flowering-time genes. *Plant Cell* 12, 885–900.

Pérez-Ruiz, R. V., García-Ponce, B., Marsch-Martínez, N., Ugartechea-Chirino, Y., Villajuana-Bonequi, M., de Folter, S., et al. (2015). XAANTAL2 (AGL14) Is an Important Component of the Complex Gene Regulatory Network that Underlies Arabidopsis Shoot Apical Meristem Transitions. *Mol. Plant* 8, 796–813. doi:10.1016/j.molp.2015.01.017.

Ratcliffe, O. J., Amaya, I., Vincent, C. A., Rothstein, S., Carpenter, R., Coen, E. S., et al. (1998). A common mechanism controls the life cycle and architecture of plants. *Dev. Camb. Engl.* 125, 1609–15.

Rédei, G. P. (1962). Supervital Mutants of Arabidopsis. *Genetics* 47, 443–60.

Samach, A., Onouchi, H., Gold, S. E., Ditta, G. S., Schwarz-Sommer, Z., Yanofsky, M. F., et al. (2000). Distinct roles of CONSTANS target genes in reproductive development of Arabidopsis. *Science* 288, 1613–1616. doi:10.1126/science.288.5471.1613.

Schwab, R., Palatnik, J. F., Riester, M., Schommer, C., Schmid, M., and Weigel, D. (2005). Specific Effects of MicroRNAs on the Plant Transcriptome. *Dev. Cell* 8, 517–527. doi:10.1016/j.devcel.2005.01.018.

Schwarz, S., Grande, A. V., Bujdoso, N., Saedler, H., and Huijser, P. (2008). The microRNA regulated SBP-box genes SPL9 and SPL15 control shoot maturation in Arabidopsis. *Plant Mol. Biol.* 67, 183–95. doi:10.1007/s11103-008-9310-z.

Shannon, S., and Meeks-Wagner, D. R. (1991). A Mutation in the Arabidopsis TFL1 Gene Affects Inflorescence Meristem Development. *Plant Cell* 3, 877–892. doi:10.1105/tpc.3.9.877.

Simon, R., Igeño, M. I., and Coupland, G. (1996). Activation of floral meristem identity genes in Arabidopsis. *Nature* 384, 59–62. doi:10.1038/384059a0.

Smith, H. M. S., Campbell, B. C., Hake, S., Colasanti, J., Sundaresan, V., Mouradov, A., et al. (2004). Competence to respond to floral inductive signals requires the homeobox genes PENNYWISE and POUND-FOOLISH. *Curr. Biol. CB* 14, 812–7. doi:10.1016/j.cub.2004.04.032.

Smith, H. M. S., and Hake, S. (2003). The interaction of two homeobox genes, BREVIPEDICELLUS and PENNYWISE, regulates internode patterning in the Arabidopsis inflorescence. *Plant Cell* 15, 1717–27. doi:10.1105/TPC.012856.

Telfer, A., Bollman, K. M., and Poethig, R. S. (1997). Phase change and the regulation of trichome distribution in Arabidopsis thaliana. *Development* 124, 645–654.

Wang, J.-W., Czech, B., and Weigel, D. (2009). miR156-regulated SPL transcription factors define an endogenous flowering pathway in Arabidopsis thaliana. *Cell* 138, 738–49. doi:10.1016/j.cell.2009.06.014.

Wang, J.-W., Schwab, R., Czech, B., Mica, E., and Weigel, D. (2008). Dual effects of miR156-targeted SPL genes and CYP78A5/KLUH on plastochron length and organ size in Arabidopsis thaliana. *Plant Cell* 20, 1231–43. doi:10.1105/tpc.108.058180.

Weigel, D., Alvarez, J., Smyth, D. R., Yanofsky, M. F., and Meyerowitz, E. M. (1992). LEAFY controls floral meristem identity in Arabidopsis. *Cell* 69, 843–59.

Weigel, D., and Nilsson, O. (1995). A developmental switch sufficient for flower initiation in diverse plants. *Nature* 377, 495–500. doi:10.1038/377495a0.

Willmann, M. R., and Poethig, R. S. (2011). The effect of the floral repressor FLC on the timing and progression of vegetative phase change in Arabidopsis. *Dev. Camb. Engl.* 138, 677–85. doi:10.1242/dev.057448.

Wilson, R. N., Heckman, J. W., and Somerville, C. R. (1992). Gibberellin Is Required for Flowering in Arabidopsis thaliana under Short Days. *PLANT Physiol.* 100, 403–408. doi:10.1104/pp.100.1.403.

Wu, G., Park, M. Y., Conway, S. R., Wang, J.-W., Weigel, D., and Poethig, R. S. (2009). The sequential action of miR156 and miR172 regulates developmental timing in Arabidopsis. *Cell* 138, 750–9. doi:10.1016/j.cell.2009.06.031.

Wu, G., and Poethig, R. S. (2006). Temporal regulation of shoot development in Arabidopsis thaliana by miR156 and its target SPL3. *Dev. Camb. Engl.* 133, 3539–47. doi:10.1242/dev.02521.

Xu, M., Hu, T., Zhao, J., Park, M.-Y., Earley, K. W., Wu, G., et al. (2016). Developmental Functions of miR156-Regulated SQUAMOSA PROMOTER BINDING PROTEIN-LIKE (SPL) Genes in Arabidopsis thaliana. *PLoS Genet.* 12, e1006263–e1006263. doi:10.1371/journal.pgen.1006263.

Yamaguchi, A., Wu, M.-F., Yang, L., Wu, G., Poethig, R. S., and Wagner, D. (2009). The microRNA-regulated SBP-Box transcription factor SPL3 is a direct upstream activator of LEAFY, FRUITFULL, and APETALA1. *Dev. Cell* 17, 268–78. doi:10.1016/j.devcel.2009.06.007.

Yant, L., Mathieu, J., Dinh, T. T., Ott, F., Lanz, C., Wollmann, H., et al. (2010). Orchestration of the floral transition and floral development in Arabidopsis by the bifunctional transcription factor APETALA2. *Plant Cell* 22, 2156–70. doi:10.1105/tpc.110.075606.

Yu, H., Ito, T., Wellmer, F., and Meyerowitz, E. M. (2004). Repression of AGAMOUS-LIKE 24 is a crucial step in promoting flower development. *Nat. Genet.* 36, 157–161. doi:10.1038/ng1286.

Yu, H., Xu, Y., Tan, E. L., and Kumar, P. P. (2002). AGAMOUS-LIKE 24, a dosage-dependent mediator of the flowering signals. *Proc. Natl. Acad. Sci. U. S. A.* 99, 16336–41. doi:10.1073/pnas.212624599.

Yu, L., Patibanda, V., and Smith, H. M. S. (2009). A novel role of BELL1-like homeobox genes, PENNYWISE and POUND-FOOLISH, in floral patterning. *Planta* 229, 693–707. doi:10.1007/s00425-008-0867-1.
